# A novel Fontan Y-graft for interrupted inferior vena cava and azygos continuation

**DOI:** 10.1093/icvts/ivac001

**Published:** 2022-02-03

**Authors:** S Samaneh Lashkarinia, Murat Cicek, Banu Kose, Mohammad Rezaeimoghaddam, Emine Hekim Yılmaz, Numan Ali Aydemir, Reza Rasooli, Sercin Ozkok, Nurgul Yurtseven, Hasan Erdem, Kerem Pekkan, Ahmet Sasmazel

**Affiliations:** 1 Department of Mechanical Engineering, Koc University, Istanbul, Turkey; 2 Department of Cardiovascular Surgery, Dr. Siyami Ersek Thoracic and Cardiovascular Surgery Training and Research Hospital, Istanbul, Turkey; 3 Department of Biomedical Engineering, Istanbul Medipol University, Istanbul, Turkey; 4 Research Hospital Radiology Department, Medeniyet University Goztepe Training, Istanbul, Turkey; 5 Anesthesiology, Pediatric Cardiac Intensive Care Unit, Dr. Siyami Ersek Thoracic and Cardiovascular Surgery Training and Research Hospital, Istanbul, Turkey; 6 Department of Cardiovascular Surgery, Kosuyolu Yuksek Ihtisas Training and Research Hospital, Istanbul, Turkey

**Keywords:** Total cavopulmonary connection, Computational fluid dynamics, Y-graft, Fontan procedure, Pre-surgical planning, Single ventricle, Hemodynamics, Extracardiac, Intratrial, Patient-specific analysis

## Abstract

**OBJECTIVES:**

To evaluate the hemodynamicdynamic advantage of a new Fontan surgical template that is intended for complex single-ventricle patients with interrupted inferior vena cava-azygos and hemi-azygos continuation. The new technique has emerged from a comprehensive pre-surgical simulation campaign conducted to facilitate a balanced hepatic flow and somatic Fontan pathway growth after Kawashima procedure.

**METHODS:**

For 9 patients, aged 2 to18 years, majority having poor preoperative oxygen saturation, a pre-surgical computational fluid dynamics customization is conducted. Both the traditional Fontan pathways and the proposed novel Y-graft templates are considered. Numerical model was validated against *in vivo* phase-contrast magnetic resonance imaging data and *in vitro* experiments.

**RESULTS:**

The proposed template is selected and executed for 6 out of the 9 patients based on its predicted superior hemodynamic performance. Pre-surgical simulations performed for this cohort indicated that flow from the hepatic veins (HEP) do not reach to the desired lung. The novel Y-graft template, customized via a right- or left-sided displacement of the total cavopulmonary connection anastomosis location resulted a drastic increase in HEP flow to the desired lung. Orientation of HEP to azygos direct shunt is found to be important as it can alter the flow pattern from 38% in the caudally located direct shunt to 3% in the cranial configuration with significantly reversed flow. The postoperative measurements prove that oxygen saturation increased significantly (*P*-value = 0.00009) to normal levels in 1 year follow-up.

**CONCLUSIONS:**

The new Y-graft template, if customized for the individual patient, is a viable alternative to the traditional surgical pathways. This template addresses the competing hemodynamic design factors of low physiological venous pressure, high postoperative oxygen saturation, low energy loss and balanced hepatic growth factor distribution possibly assuring adequate lung development.

**Date and number of IRB approval:**

25 October 2019, 280011928-604.01.01.

**Figure:**
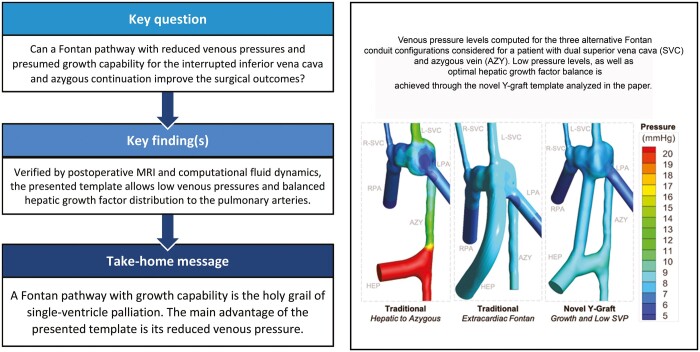

## INTRODUCTION

Left atrial isomerism is a complex syndrome in which the atria resemble left atrial morphology and is accompanied by multiple cardiac and non-cardiac anomalies [1]. In almost all cases, the inferior vena cava (IVC) is non-existent at the right atrium connection and the azygos (AZY) connection to the superior vena cava (SVC) is prominent. Currently, the most common single-ventricular surgery is the Kawashima procedure, whereby all systemic venous return is directed to the pulmonary artery (PA) with a cavopulmonary anastomosis procedure, except for the hepatic veins (HEP) [2]. The main disadvantage of this procedure is the absence of hepatic inhibitory growth factors in the pulmonary circulation, resulting in pulmonary arteriovenous fistulas with declining tissue oxygenation. For this complex disease, the traditional surgical templates reported in the literature include an intra-atrial [3] or extracardiac graft [4], and a direct shunt from the HEP to AZY or one of the PA branches [5, 6].

In a more recent study, de Zélicourt *et al.* [7] combined virtual surgery and numerical simulations to identify potential surgical strategies for single-ventricle patients with interrupted IVC. The research findings indicated that for single SVC patients, intra-atrial baffles and extracardiac conduits resulted in problematic flow distribution on the basis that even a small left or right offset led to highly preferential HEP flow direction to the associated lung. Alternative surgical templates, including bifurcated graft (Y-graft) connected to PA branches or connected to AZY and a PA branch (H connection) and direct HEP to AZY shunts, promote mixing of HEP flow with the rest of venous flow. Y-shape Fontan total cavopulmonary connection shunts were introduced in an earlier study [8], which presented an optimized split-anastomosis Fontan connection directly to both the left pulmonary artery (LPA) and right pulmonary artery (RPA) branches to avoid the dissipative inflow collision. The research findings showed that Y-grafts significantly improve hemodynamicdynamic performance at higher cardiac outputs [8], which motivated further design and feasibility studies of novel surgical templates. A number of studies have examined the use of Y-graft modification for the Fontan operation using computational blood flow simulations and patient-specific models [9–11]. The increased energy efficiency, lower blood pressures and improved hepatic factor distribution observed in comparison to traditional Fontan templates indicated that the Y-graft could be a promising modification for the Fontan procedure [12]. Previous studies have also shown that creating preoperative anatomy and blood flow numerical simulations helps to identify the best surgical approach for each patient individually [13]. A recent study of 60 single-ventricle patients who underwent Fontan surgery [14] showed that for commercially available Y-graft procedures, hepatic flow distribution performance depends on multiple factors including pulmonary flow distribution, pulmonary artery stenosis and SVC positioning. Y-grafts can result significantly higher resistance than the extracardiac conduits because of their conduit geometry, which calls for optimal Y-graft design [14]. Research continues to examine emerging surgical techniques which are designed to improve the outcomes of the Fontan surgery for interrupted IVC and AZY continuation [15, 16].

In the present study, we developed a new technique for Fontan Y-graft surgery for interrupted IVC and AZY continuation. In this configuration, one branch is connected to the PA bed and the other branch is connected to AZY. The novel Y-graft is tested through an unbiased pre-surgical computational planning campaign to guarantee the optimum HEP distribution as well as hydrodynamic energy loss to improve exercise capacity [17]. Postoperative follow-up magnetic resonance imaging (MRI) scans were acquired to verify its ability to promote more favourable HEP flow distribution and the possibility for adequate native AZY growth as the infant or adolescent patient grows.

## MATERIALS AND METHODS

### Patient data

A cohort of 9 patients from the ages of 2–18 years with interrupted IVC and azygos (AZY) and hemi-AZY continuation were recruited for this study (Table 1), in accordance with the institutional review board approval process of the Dr Siyami Ersek Thoracic and Cardiovascular Surgery Research Hospital (25 October 2019: Approval Number 280011928-604.01.01). Formal written consent is obtained from study participants and their parent/guardian. Three-dimensional pre-surgical anatomical reconstructions are performed including the surgical landmarks of the ventricle, aorta and hepatic veins (provided in Supplementary Material, *S1*). Patients were recruited consequently, and this order is available in Table 1. Patients regularly listed for surgery is included with no exclusion criteria.

**Table 1:** Demographic data of the patient cohort, information about the tested virtual surgery configurations, pre- and postoperative hepatic flow splits from computational fluid dynamics simulations and clinically measured pre- and postoperative oxygen saturation levels

Patient	Age	BSA (m^2^)	Weight (kg)	sex	Bilateral SVC	Number of Surgical alternatives simulated	Number of Y-graft models simulated	Selected graft type	**Selected graft diameter (mm)** PA branch/AZY branch	Pre-surgery hepatic flow		Post-op Hepatic flow		Pre-op Oxygen sat. (%) a	Post-op Oxygen sat. (%) a
										Total (%)	LPA/RPA	Total (%)	LPA/RPA		
1	6	1.1	43	M	Yes hemi-azy	9	4	Novel Y-graft	10/14	46	15/31	100	31/69	73	91
2	14	1.2	37	F	Yes hemi-azy	3	3	Novel Y-graft	12/12	100	0/100	100	38/62	74	93
3	11	1.19	24	M	No	6	1	Novel Y-graft	10/14	0	0/0	100	33/67	80	89
4	13	1.3	31	F	Yes hemi-azy	4	1	Novel Y-graft	10/14	29	27/2	100	27/73	79	92
5	18	1.4	48	F	Yes	5	3	Novel Y-graft	10/14	12	7/5	100	39/61	80	92
6	7	0.7	17	F	No	3	2	Novel Y-graft	10/14	46	0/46	100	55/45	75	89
7	9	1.2	30	F	No	9	1	HEP to AZY	15	76	40/36	100	43/57	88	96
8	6	0.4	18	M	No	3	1	Extra-cardiac	14			100	67/33	90	95
9	2	0.3	10	F	No	4	2	Extra-cardiac	14			100	50/50	87	94

a Oxygen saturation increased statically, but significantly (*P*-value = 0.00009) to normal levels in 1-year postoperative follow-up. Pre-surgery hepatic flow is the part that is delivered to the lungs. Pre-surgery three-dimensional reconstructions and CFD results of all surgical alternatives simulated are provided in Supplementary Material, *S1*. In this cohort, all patients were at post-Glenn stage at the time of computational planning. Patients are recruited in the following order: 7-1-3-4-2-5-6-8-9.

AZY: azygos vein; CFD: computational fluid dynamics; HEP: hepatic veins; LPA: left pulmonary artery; PA: pulmonary artery; RPA: right pulmonary artery; SVC: superior vena cava; BSA: Body surface area.

### Computational fluid dynamics

Computational fluid dynamics (CFD) simulation methodology was identical to our earlier studies dedicated to Fontan patients [18, 19]. Pre-surgical three-dimensional anatomy, phase-contrast PC-MRI flow boundary conditions and cardiac catheterization data are used to estimate resistant boundary conditions specified downstream of pulmonary arteries. Further details of MRI scanning protocol are provided in the Supplementary Material, *S2*. Post-op MRI scans are performed for CFD model verification for the recruited patients. For additional numerical model verification, 1 surgical model (Patient 7) was stereo-lithographically printed and tested in a mock-up Fontan flow loop. In this experiment, hepatic flow was measured through dye concentrations and found to be within reasonable agreement having a maximum of 10% error (see Supplementary Material, *S2* for more details). This order of accuracy is acceptable compared to significantly larger errors involving in patient image data.

### Virtual surgery

Using CFD simulations a comprehensive patient-specific customization is achieved during the pre-surgical planning phase. In addition to the novel Y-graft, hemodynamicdynamic performance of traditional surgical templates (intra- and extracardiac) is evaluated and considered as a preferred alternative (Supplementary Material, *S2*). For example, for Patient 1 (Table 1), 9 different surgical configurations were virtually generated in the computer, using the in-house sketch-based computer interface that we developed in a previous study [20]. The second surgical configuration alternative designed for Patient 1 is annotated as *Patient 1_2* in this manuscript*.* Simulated baffle types and dimensions are provided (Table 2). As such, for Patient 2 (Table 1) who required a redo Fontan operation, two (2) surgical alternatives were generated following the same virtual surgery protocol. In order to improve pathway hemodynamicdynamics over the previous failed Fontan configuration, we applied our novel Y-graft surgical template in this patient as well. Configurations designed for this patient included a direct shunt between AZY and the previously implanted extracardiac baffle, which was anastomosed with different angles.

**Table 2:** Specifications of tested models and summary of computational fluid dynamics simulation results for Patient 1

Case name	Baffle type	D (m)	Baffle offset (mm)	SVC offset (mm)	Hepatic flow distribution (%)		Total flow distribution (%)				Power loss (mW)
					Left	Right	Left		Right		
Pre-surgery											
*1_Pre*	No baffle				15	31		22		78	25
Surgical planning scenarios											
*1_1*	Extracardiac	14	Left		86	14		44		56	22
*1_2*	Extracardiac	14			26	74		43		57	19.9
*1_3*	HEP to AZY	12			52	48		48		52	113.4
*1_4*	HEP to AZY	14			48	52		47		53	98.6
*1_5*	HEP to AZY	14		13/Right	64	36		46		54	100.9
*1_6*	Y-graft				15	85		41		59	23.9
	HEP to AZY	14									
	HEP to PA	12									
*_7*	Y-graft		8/ Left		90	10		47		53	28
	HEP to AZY	14									
	HEP to PA	12									
*1_8*	Y-graft (Selected)				31	69		44		56	30
	HEP to AZY	14									
	HEP to PA	10									
*1_9*	Y-graft				27	73		43		57	35.1
	HEP to AZY	14									
	HEP to PA	8									
Growth estimation (scale factor= 1.2)											
*1_ G1*	Y-graft (Selected)				35	65		48		52	21
	HEP to AZY	14									
	HEP to PA	10									
*1_ G2*	Y-graft				30	70		47		53	23.2
	HEP to AZY	14									
	HEP to PA	8									
Post-surgery											
*1_Post*	Y-graft				32	68		58		42	15
	**HEP to AZY**	14									
	**HEP to PA**	10									

Each surgical template subgroup is shown in a different. *Patient 1_7* corresponds to the seventh numerically simulated pre-surgical alternative considered for Patient 1. Two estimates of lumen growth (*G1* and *G2*) are also tested to verify long-term hemodynamicdynamics. Computational simulation results of the actual postoperative configuration obtained from post-op MRI is also provided (*Patient 1_Post*).

AZY: azygos vein; HEP: hepatic veins; MRI: magnetic resonance imaging; PA: pulmonary artery; SVC: superior vena cava.

To the best our knowledge, *pre-surgical* hepatic flow distribution is estimated, first-time in literature, by computing the intracardiac flows to evaluate the relative benefits of the surgery. In order to further study of the effect of postoperative somatic vascular growth, a virtual growth-state model was also created from the reconstructed preoperative geometry by scaling 1.2 fold of its original size, based on post-op MRI findings. Which also represents the diameter enlargement of PA branches in 1-year somatic growth [21] while the baffle diameter was kept constant (Table 2—growth estimation subgroup). The finally executed surgical configuration is selected based on the hepatic flow split, pulmonary flow split and energy loss performance with roughly equal weighting.

### Surgical techniques

**Figure 1:**
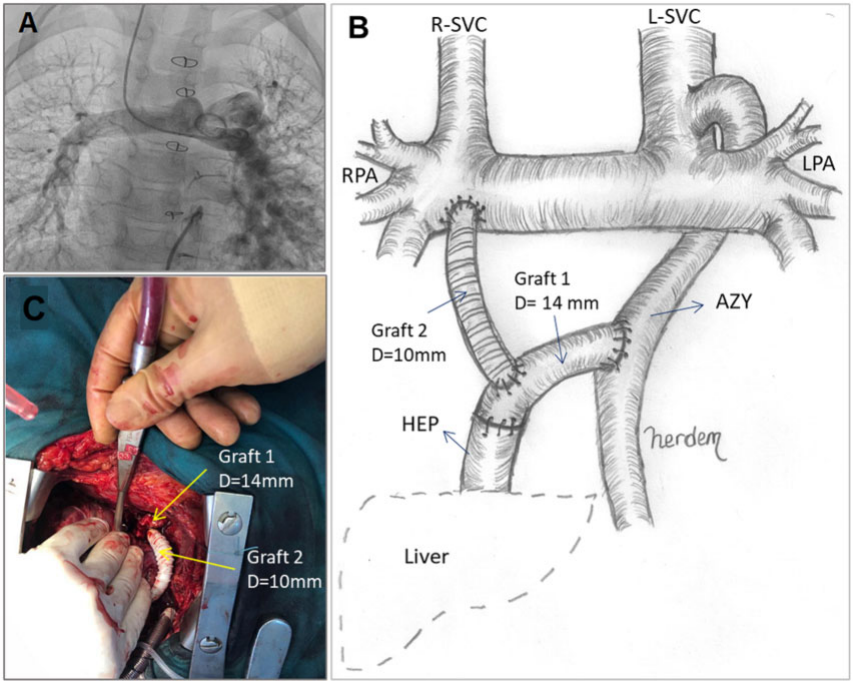
(**A**) Pulmonary angiogram of Patient 1, displaying left pulmonary arteriovenous fistula. (**B**) Novel Y-graft template for directing HEP flow to AZY and PA bed. (**C**) Surgical layout during the implantation of two 10–14 mm Y-grafts. AZY: azygos vein; HEP: hepatic veins; PA: pulmonary artery.

After a redo sternotomy procedure was performed on all 9 patients, the aorta, PAs, cardiac mass and HEP-draining right-sided atrium were dissected. Following cardiopulmonary bypass, the cardiac apex was elevated, and an oblique incision was made on the posterior pericardium. Taking care not to sever the phrenic nerve, the posterior mediastinum was exposed just above the diaphragm through the pericardium. The AZY was dissected, and a C-clamp was applied. For Patient 1, a 14-mm polytetrafluoroethylene tube graft was anastomosed between HEP and AZY in an end-to-side manner on the AZY side as well as an end-to-end manner on the HEP side. In addition, a 10-mm ringed (in order to avoid kinking) polytetrafluoroethylene tube graft was anastomosed between the HEP to AZY graft and right side of the pulmonary bed in an end-to-side manner on both sides using 6-0 prolene continuous suture (Fig. 1B). Intraoperative surgical implementation for Patient 1 is shown in Fig. 1C. For Patient 2, a 14-mm polytetrafluoroethylene tube was anastomosed between AZY and previously implanted extracardiac graft in an end-to-side manner on both sides.

Following the surgery oxygen saturation levels of all patients are recorded within 1 year. Pre- and postoperative saturation levels are compared using the paired *t*-test to test the null hypothesis, H0, which states that the means of Oxygen saturation levels of pre-op and post-op do not differ significantly. Two-tailed *P*-value is compared with alpha level of 0.05. We compared outcome with standard Fontan patients though this patient group is unique.

## RESULTS

The CFD simulation results of all surgical alternatives considered are provided in the Supplementary Material, *S1*. For brevity, only Patients 1 and 2 are discussed in detail to illustrate the hemodynamicdynamic characteristics of the new Y-graft.

### Hepatic flow split before surgery

**Figure 2:**
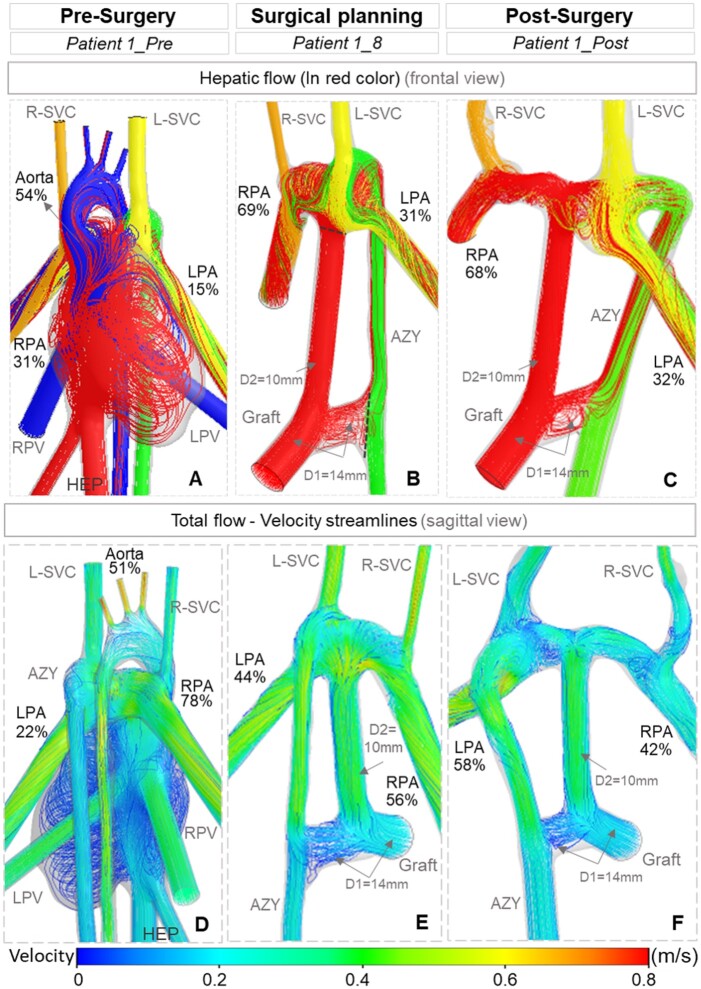
Computational fluid dynamics simulations of (**A, D**) pre-surgery, (**B, E**) selected eighth surgical alternative (*Patient 1_8*) and (**C, F**) post-surgery model. First row shows the streamlines of inflows in different colours (**A–C**). Hepatic flow split is shown in red colour. Second row indicates the velocity streamlines and total venous flow splits to the lungs (**D–F**). Quantified flow splits to the lungs are reported in percentages.

The hemodynamicdynamics of the pre-surgical state is often ignored in the computational hemodynamicdynamic surgical planning studies. Pre-surgery condition is highly variable for this patient group, involve complex intracardiac flows. Here, it served as a *baseline* to estimate improvements possible by the optimal surgical design. hemodynamicdynamic pre-surgery simulations for Patient 1 indicated that more than half (54%) of the HEP flow went directly to the aorta without entering the lungs (Table 2—*Patient 1_Pre* simulation). We predicted that the LPA received only 15% of the total HEP flow (Fig. 2A) before the surgery. Larger cross-sections of the RPA compared to the LPA correlate with right lung preferential venous flow (78%; Fig. 2D).

### Traditional pathway designs for Patient 1

For Patient 1, 9 alternative surgical configurations are simulated. These results are summarized in Table 2 (for other patients, please refer to the Supplementary Material, *S1*). Two extracardiac configurations with 14-mm diameter size were tested in *Patient 1_1* and *Patient 1_2* cases. Positioning the graft anastomosis to the PA at the left side of PA in *Patient 1_1* led to 86% biased HEP flow to the left lung. Alternatively, placing the extracardiac conduit at the middle of the PA (*Patient 1_2*) was associated with the promotion of 74% HEP flow to the right lung. Total venous flow to both PA branches was balanced in both extracardiac cases with only 3–4% flow split differences. In terms of energy loss in the pulmonary flow, extracardiac models led to the lowest power-loss levels (19.9–22 mW) among all of the simulations (Table 2).

**Figure 3:**
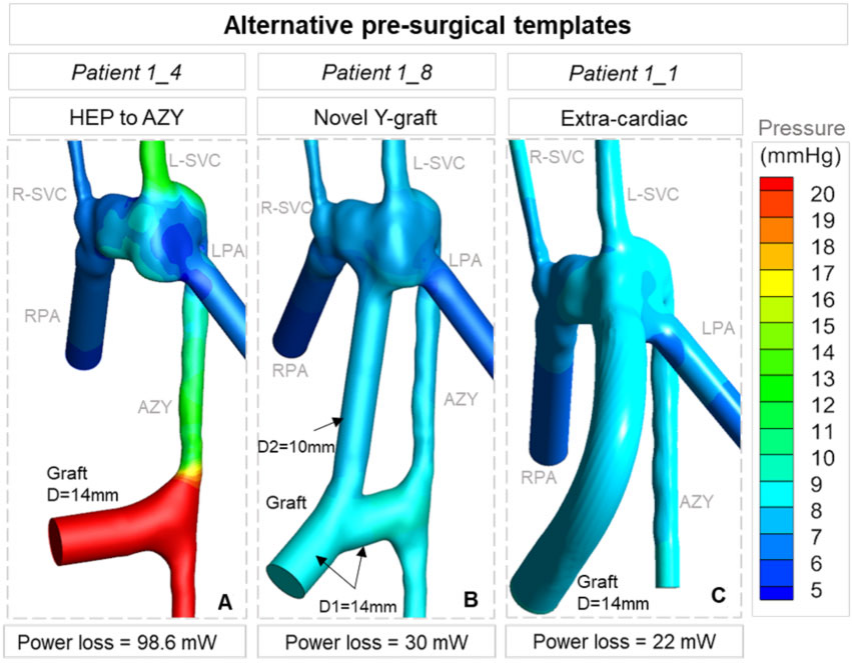
The general characteristics of the 3 main surgical templates are illustrated through numerical simulations. The extracardiac template (*Patient 1_1*) had the lowest pressure levels but with very poor HEP flow split (86/14). While the direct HEP to AZY shunt (*Patient 1_4*) would allow for balanced HEP flow (48/52), it led to unacceptably high venous pressure levels. The new modified Y-graft (*Patient 1_8*) demonstrated optimal values for hepatic flow (31/69) and venous pressure. Patient 1_8 corresponds to the eighth surgical alternative of the first patient. AZY: azygos vein; HEP: hepatic veins.

In comparison, the HEP to AZY direct shunt models showed a favourable distribution of both HEP and venous flow to the lungs in *Patient 1_3* and *Patient 1_4* cases (48% and 52% flow splits, respectively). High levels of energy loss (98.6–113.4 mW) were the main drawbacks in these graft types compared to other surgical templates. As shown in Fig. 3, a 20-mmHg pressure level was observed for the graft for *Patient 1_4* (Fig. 3A), while this value dropped to 8–9 mmHg for *Patient 1_1* and *Patient 1_8* cases (Fig. 3B and C, respectively). In the simulation model for *Patient 1_5*, a 14-mm HEP to AZY direct shunt with a 13-mm Kawashima anastomosis movement to the right was tested. Kawashima anastomosis movement to the right side disturbed the HEP flow balance by increasing the HEP flow to the LPA (64%) compared to *Patient 1_4*.

### Geometrical features of the novel Y-graft

#### Baffle size

**Figure 4:**
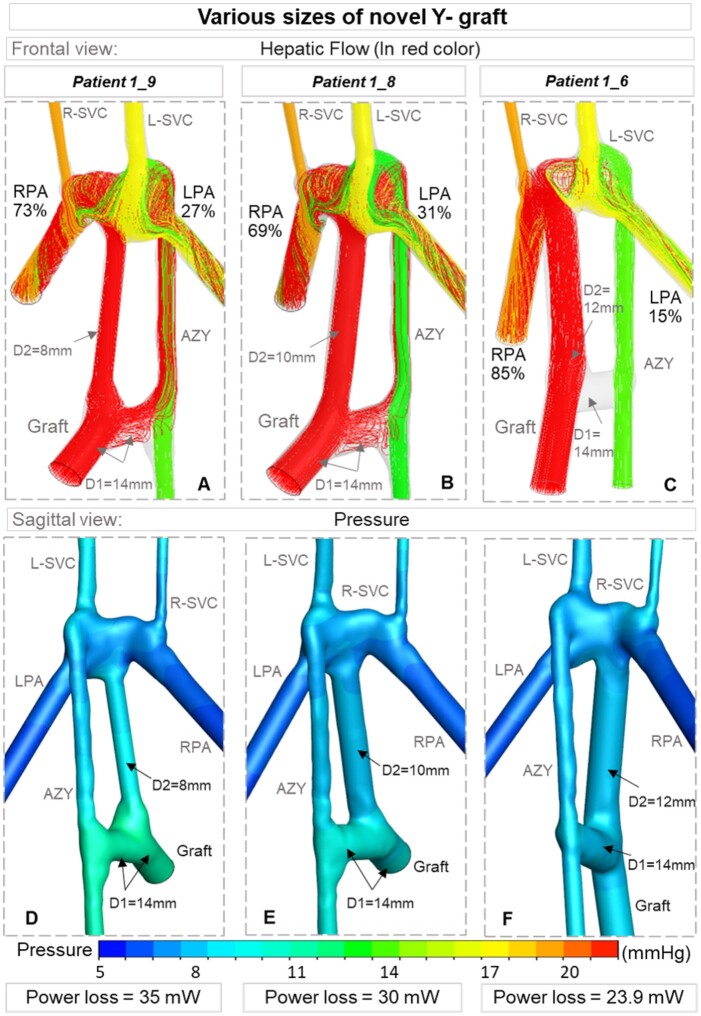
Three Y-graft configurations (Table 2) are compared in terms of HEP flow splits (shown in red colour, A, B and C), pressure level (D, E and F, respectively) and power loss. The HEP to AZY branch is 14 mm in all models and the HEP to PA branch size varies between 8 and 12 mm. Flow splits to the lungs are computed from computational fluid dynamics simulations and reported in percentages. AZY: azygos vein; HEP: hepatic veins.

In the novel Y-graft models, which were customized through surgical alternatives *Patient 1_6 to Patient 1_9*, the size of the HEP to AZY branch was 14 mm, whereas the size of the HEP to PA branch varied between 8 and 12 mm (Fig. 4). The two Y-graft configurations of 12–14 mm were tested in *Patient 1_6* and *Patient 1_7* models (Table 2). In *Patient 1_7*, an offset of 8 mm towards the LPA led to 90% HEP flow to the left lung, while placing the graft in the middle of the PA in *Patient 1_6* led to 85% HEP flow to the RPA. Interestingly for both configurations, the HEP to AZY branch did not direct any blood to AZY and hence no flow was observed in this branch (Fig. 4C). The small size of the HEP to PA branch (8 mm) in *Patient 1_9* improved HEP flow split to the LPA from 15% in *Patient 1_6* to 27% in *Patient 1_9* (Fig. 4A). However, it was also associated with 45% increase in power loss compared to *Patient 1_6* (Table 2). The findings indicate that the optimum HEP to PA branch size was around 10 mm diameter (*Patient 1_8*) in the Y-graft configurations, which led to 31% and 69% flow splits to the LPA and RPA, respectively (Fig. 4B). Furthermore, we found that power loss was 5 mW less in this model compared to *Patient 1_9*. Therefore, the novel Y-graft model (*Patient 1_8*) was adopted as the actual surgical template as it maintained the beneficial balanced hepatic flow of a single HEP to AZY shunt, but with low venous pressure levels.

#### Orientation of the direct shunt

**Figure 5:**
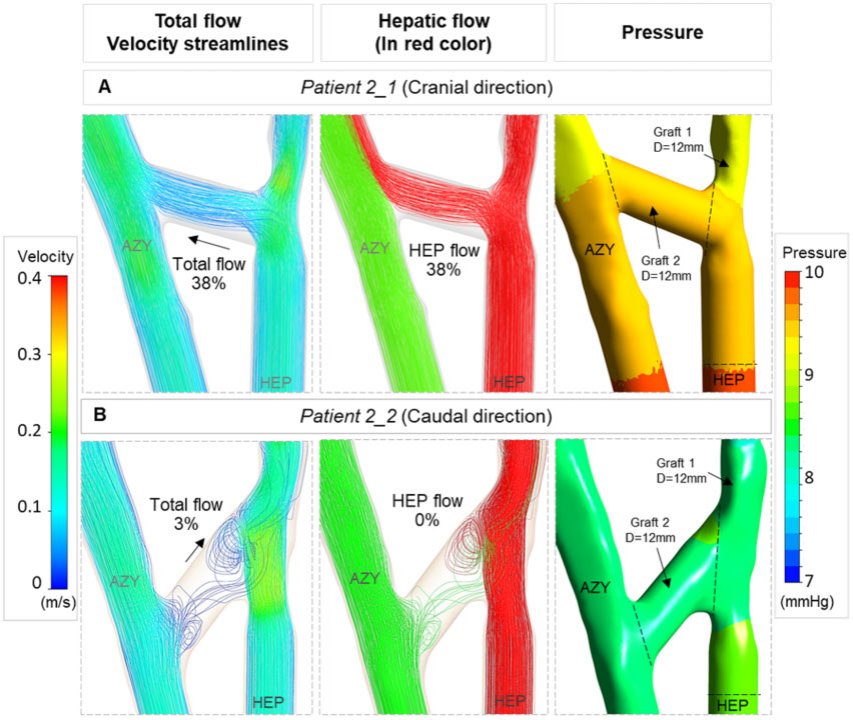
Effect of branch angle of the HEP to AZY graft (Graft 2) in flow pattern illustrated via numerical simulation results. First column indicates the velocity streamlines and total venous flow splits to the lungs. Second column shows the streamlines of inflows in different colours. Hepatic flow split is shown in red colour. Third column shows the computed pressure level. Quantified flow splits to the lungs are reported in percentages. (**A**) Orienting the HEP to AZY graft (Graft 2) in cranial direction was associated with directing 38% HEP flow to AZY. (**B**) Graft 2 in caudal direction from HEP to AZY failed to conduct the HEP flow to AZY and 3% retrograde flow from AZY to extracardiac baffle was observed. AZY: azygos vein; HEP: hepatic veins.

In the surgical configurations generated for the corrective surgery case (Patient 2), the direction of the 14-mm diameter direct shunt between extracardiac baffle to AZY was located in both the caudal (*Patient 2_1—*Fig. 5A) and cranial (*Patient 2_2—*Fig. 5B) directions. With the same anatomy and flow conditions, the flow pattern of the direct shunt changed significantly from 38% HEP to AZY flow in the cranially located graft compared to 3% in the caudal configuration with significantly reversed flow.

### Novel hepatic Y-graft performance

In the *Patient 1_8* case, HEP flow to the LPA was elevated 107% compared to the pre-surgery model (*Patient 1_Pre*). Total venous flow in the selected virtual surgery model was predicted to be more balanced at 44% and 56% for the LPA and RPA, respectively (Fig. 2E). In the real post-surgery model (*Patient 1_Post*), total venous flow split to the lungs was balanced, but the venous flow to the LPA was 16% less than the RPA (Fig. 2F). Predicted HEP flow split to the lungs in *Patient 1_8* was in a good agreement with the post-surgery model (*Patient 1_Post*) anatomy and clinical results, with a prediction error less than 3% (Fig. 2B).

### Influence of presumed postoperative growth

In the postoperative growth estimation subgroup (Table 2), the effects of growth were simulated as 20% lumen cross-section enlargement in the native vessels only. In *Patient 1_G1* and *Patient 1_G2* cases, which are the scaled versions of *Patient 1_8* and *Patient 1_9* cases, no significant change was observed in terms of HEP and total venous flow patterns. As expected, the power loss dropped 30% and 33% compared to the original models in *Patient 1_G1* and *Patient 1_G2* cases, respectively, due to larger lumen size. During the early postoperative period, the native AZY pathway is the resistance bottleneck of the proposed template, since HEP to AZY shunt diameter is selected to be as large as possible. The total cavopulmonary connection resistance is expected to decrease further at late post-op due to the presumed growth of the high flow native AZY pathway (Supplementary Material, *S3*).

## DISCUSSION

Previous studies have shown that being able to control antegrade pulmonary flow in the Kawashima procedure can prevent or delay the development of pulmonary arteriovenous fistulas by exposure of the pulmonary bed to hepatic flow [22]. However, this also leads to the development of large veno-venous collaterals that redirect the pulmonary flow to the heart and high blood pressure in the PA. Therefore, retrograde flow patterns in AZY may occur in some cases due to the antegrade pulmonary flow [23]. We identified the same condition in Patient 1 in the present study, who had a high level of blood pressure in the left side of the pulmonary vascular bed due to the antegrade flow associated with pulmonary arteriovenous fistula formation of the left lung.

Among the various simulated surgical configurations for Patient 1, a direct HEP to AZY shunt provided the most balanced hepatic and pulmonary flow to the lungs. However, directing HEP blood through the narrow AZY causes very high-pressure levels in the graft and leads to elevated energy loss. In this respect, a conventional Y-graft with branches anastomosed to the LPA and RPA may be a promising design with the potential to reduce energy loss and improve hepatic flow distribution [10, 24].

In the present study, we present a modified, novel Y-graft configuration in particular for interrupted IVC and AZY continuation by combining a direct HEP to AZY shunt and a conventional Y-graft model. Previous studies introduced the Y-shape and H-connection graft as direct channels to split the flow from the Hepatic origin while in this template, a small-sized graft is implanted between the HEP to AZY graft and the PA bed as a collateral, which functions as a pressure release pathway and reduces the power loss in the early postoperative period when resistance in the AZY is high. Hepatic flow to the left lung can be sustained through the HEP to AZY graft while the other branch guarantees hepatic flow to the right lung. The efficacy of this novel Y-graft configuration was observed in the postoperative MRA around 1 year later, whereby the fistulas in the LPA had disappeared and both lungs had adequate perfusion. Since AZY drained to the LPA in this Patient 1, it can be interpreted that increased flow to the AZY is associated with increased total venous flow to the LPA as well. Therefore, the underdeveloped LPA that was observed in the pre-surgery scans was able to grow well and the balanced size of the LPA and RPA is recorded in postoperative data (Fig. 2C and F).

The size of the extracardiac conduit in the modified Y-graft is a critical parameter. We found that the small size (8 mm) of the Y-graft for *Patient 1_9* was associated with higher power loss and more biased HEP flow to RPA compared to the selected model (*Patient 1_8*). On the other hand, selecting a much bigger size for the extracardiac branch (14 mm) directs all of the HEP flow to the PA and no flow is directed to the HEP to AZY shunt (*Patient 1_6*). These findings indicate that the 10 mm branch size is the optimal case (*Patient 1_8*).

We also generated virtual surgery configurations for the corrective surgery case (*Patient 2_1* and *Patient 2_2*—Fig. 5) to highlight the importance of the direction of the HEP to AZY branch in our novel Y-graft configuration. The computational simulation results show that while the graft sizes were the same in 2 identical simulations (Fig. 5), the direction of the HEP to AZY shunt impacts the flow pattern and graft functionality significantly. These geometric features can be tested easily through the proposed surgical design approach.

We tried to predict the postoperative state 1 year after surgery by scaling the native vessels with a scale factor of 1.2, which considers 1.5–2 mm growth for the diameter of the PA branches (*Patient 1_G1* and *Patient 1_G2*). Comparison of the scaled Y-graft model (*Patient 1_G1*) with the clinical postoperative model (*Patient 1_Post*) indicates that even though the HEP flow splits were predicted with less than 9% error, the scaled model failed in predicting the total venous flow splits and power loss. One main reason for this discrepancy may be that in reality, actual growth is not uniform in all vessels. For example, in the *Patient 1_Post* model, 3 mm growth of AZY in diameter was significant compared to other vessels. A more elaborated tissue growth model like the one being used in our ongoing work [25] can improve the prediction of long-term postoperative hemodynamicdynamics for these patients. The native lumen growth of the inferior venous return (hepatic + azygous) is more critical than the Glenn/Kawashima because as the patient grows the per cent of cardiac output that SVC has decreases significantly (50% newborn to 20% older adult) and the remaining flow split drains to the inferior venous vessels. Good size baffles can handle this for the general patient population, but for the complex cohort presented here a patient-specific optimization results in a better outcome. Low flow from hepatic vein, the need for better exercise capacity and reduced thrombosis requires precise graft size adjustment.

For this patient group, the partitioned hepatic venous return, colliding and flow recirculation zones (as in Fig. 5B) can pose a high risk of conduit thrombosis [26, 27]. While a detailed quantitative patient-specific pre-surgical hemodynamiclysis assessment is justified for the selected surgical configuration, in our approach, three-dimensional flow streamlines are examined qualitatively, but in detail, for potentially problematic flow patterns due to the short surgical timeline. Postoperative follow-up did not indicate hemodynamiclysis related problems in this patient group. All patients did well with standard anticoagulation protocol.

It is important to highlight that the novel Y-graft template was examined in virtual surgery simulations for all 9 patients together with the traditional Fontan templates (Supplementary Material, *S1*). However, no specific surgical templates are initially favoured in this unbiased approach and the novel Y-graft template was selected for actual surgery for 6 of these 9 patients (Patients 1 to 6—Table 1). Postoperative clinical measurements taken from these patients show that all surgeries were successful and improved postoperative hemodynamicdynamics were observed in all patients. Pre-surgery and postoperative MRA recordings for Patient 6 are shown in Supplementary Material, *S4* as a short video. More specifically, statistical tests carried out on the novel Y-graft cases indicates that oxygen saturation increased statically significantly to 92 ± 2% (*P*-value = 0.00009) within 1 year after surgery, reaching above the standard Fontan completion levels [28, 29].

## CONCLUSION

The precise novelty of the proposed surgical template is in its ability to significantly reduce venous pressure and its potential to facilitate vascular growth. As the native AZY lumen increase with age, there is further *potential* for improved hemodynamicdynamics. This would *possibly* lead improved quality of life and improved exercise capacity. Despite the complex long-term vascular growth and remodelling of Fontan patients, computational hemodynamicdynamic predictions agree with the *in vivo* postoperative measurements for the patient cohort in this study. Virtual surgical simulations found to be effective in precise patient-specific optimization of HEP factor split and pulmonary perfusion, which leads to significantly better postoperative oxygen saturation levels. Proposed template can be adopted due to its potential clinical benefits and its natural emergence from an unbiased pre-surgical computational customization campaign. Due to the variability of patient anatomy and flowrates, a computational pre-surgical customization is recommended for any surgical template type, including the proposed Y-graft, to verify the optimal hemodynamicdynamics.

## SUPPLEMENTARY MATERIAL

Supplementary material is available at *ICVTS* online.

## Supplementary Material

ivac001_Supplementary_Data

## ABBREVIATIONS

AZY: Azygos vein
CFD: Computational fluid dynamics
HEP: Hepatic veins
IVC: Inferior vena cava
L/R-SVC: Left/right branch of SVC
LPA: Left pulmonary artery
PA: Pulmonary artery
RPA: Right pulmonary artery
SVC: Superior vena cava
