# Supplementary material for: A novel Fontan Y-graft for interrupted inferior vena cava and azygos continuation
Source: Interact Cardiovasc Thorac Surg. 2022 Feb 3;34(6):1095–105. doi: 10.1093/icvts/ivac001 (PMC9159461; doi:10.1093/icvts/ivac001)
Supplement: ivac001_Supplementary_Data [file ivac001_supplementary_data.zip › ivac001-suppl_data/Supplementary Material 1.pptx]

## Slide 1
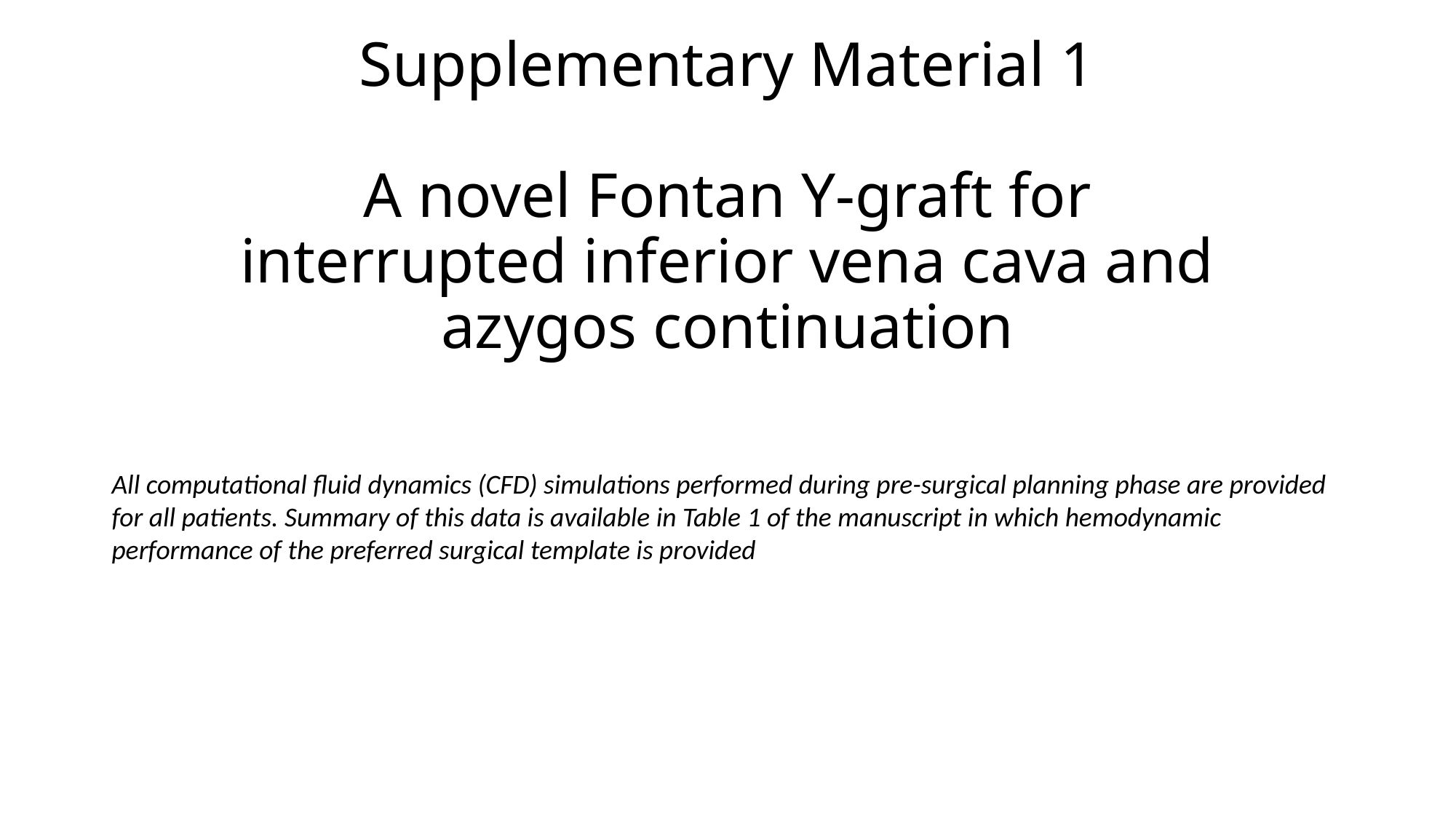

# Supplementary Material 1 A novel Fontan Y-graft for interrupted inferior vena cava and azygos continuation
All computational fluid dynamics (CFD) simulations performed during pre-surgical planning phase are provided for all patients. Summary of this data is available in Table 1 of the manuscript in which hemodynamic performance of the preferred surgical template is provided

## Slide 2
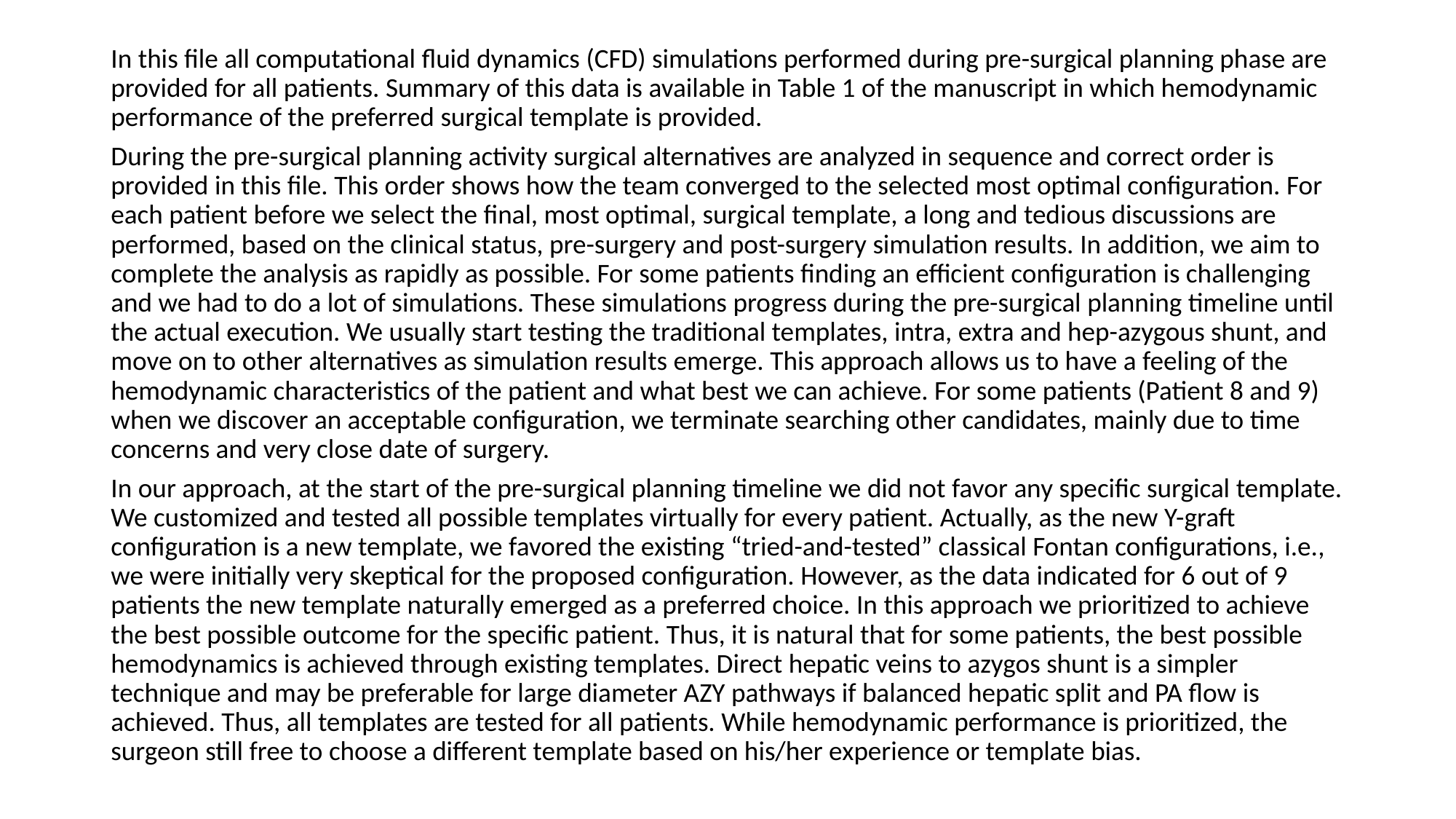

In this file all computational fluid dynamics (CFD) simulations performed during pre-surgical planning phase are provided for all patients. Summary of this data is available in Table 1 of the manuscript in which hemodynamic performance of the preferred surgical template is provided.
During the pre-surgical planning activity surgical alternatives are analyzed in sequence and correct order is provided in this file. This order shows how the team converged to the selected most optimal configuration. For each patient before we select the final, most optimal, surgical template, a long and tedious discussions are performed, based on the clinical status, pre-surgery and post-surgery simulation results. In addition, we aim to complete the analysis as rapidly as possible. For some patients finding an efficient configuration is challenging and we had to do a lot of simulations. These simulations progress during the pre-surgical planning timeline until the actual execution. We usually start testing the traditional templates, intra, extra and hep-azygous shunt, and move on to other alternatives as simulation results emerge. This approach allows us to have a feeling of the hemodynamic characteristics of the patient and what best we can achieve. For some patients (Patient 8 and 9) when we discover an acceptable configuration, we terminate searching other candidates, mainly due to time concerns and very close date of surgery.
In our approach, at the start of the pre-surgical planning timeline we did not favor any specific surgical template. We customized and tested all possible templates virtually for every patient. Actually, as the new Y-graft configuration is a new template, we favored the existing “tried-and-tested” classical Fontan configurations, i.e., we were initially very skeptical for the proposed configuration. However, as the data indicated for 6 out of 9 patients the new template naturally emerged as a preferred choice. In this approach we prioritized to achieve the best possible outcome for the specific patient. Thus, it is natural that for some patients, the best possible hemodynamics is achieved through existing templates. Direct hepatic veins to azygos shunt is a simpler technique and may be preferable for large diameter AZY pathways if balanced hepatic split and PA flow is achieved. Thus, all templates are tested for all patients. While hemodynamic performance is prioritized, the surgeon still free to choose a different template based on his/her experience or template bias.

## Slide 3
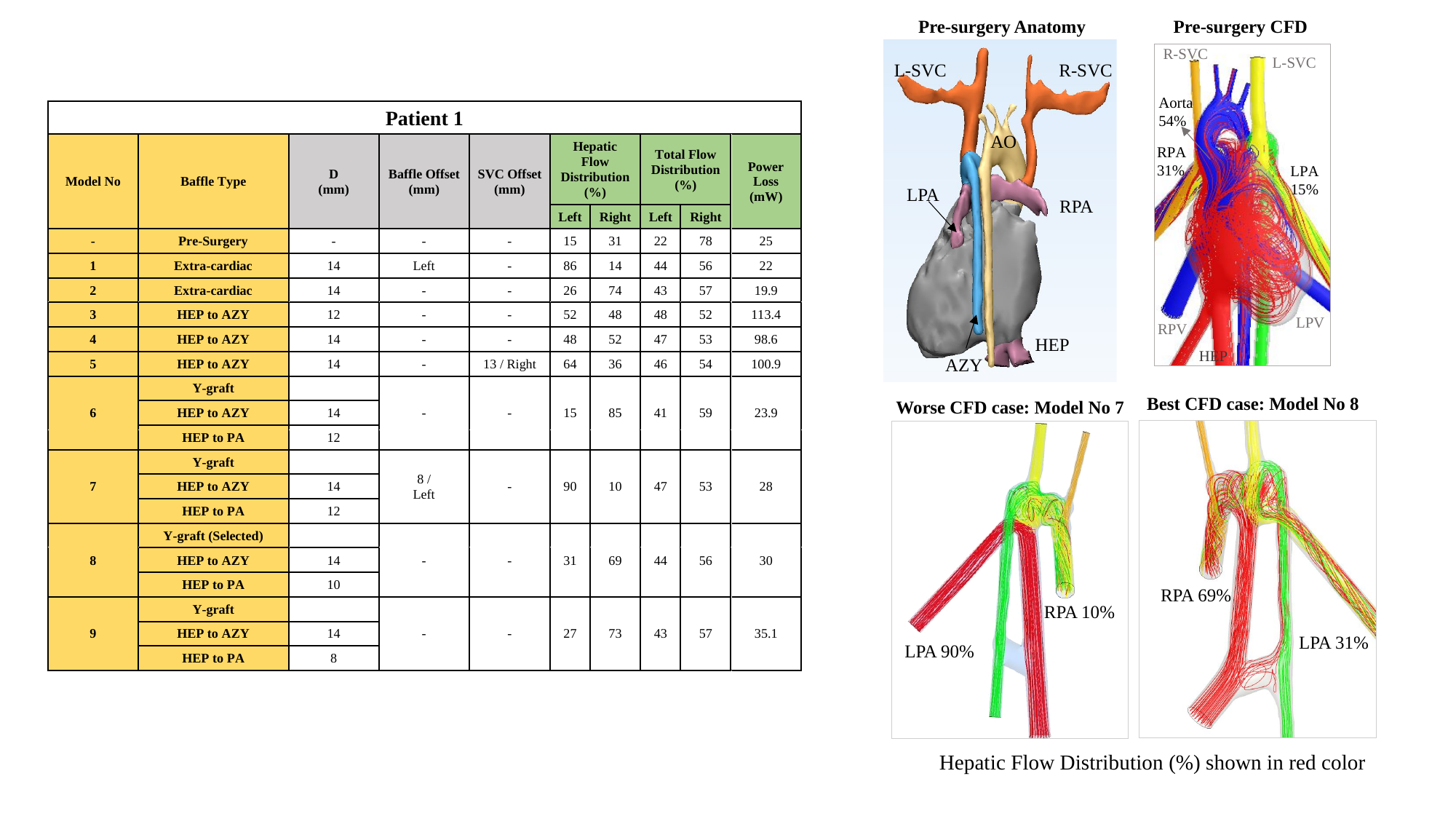

Pre-surgery Anatomy
R-SVC
L-SVC
AO
LPA
RPA
AZY
HEP
Pre-surgery CFD
R-SVC
L-SVC
Aorta
54%
RPA
31%
LPA
15%
LPV
RPV
HEP
Best CFD case: Model No 8
RPA 69%
LPA 31%
Worse CFD case: Model No 7
RPA 10%
LPA 90%
Hepatic Flow Distribution (%) shown in red color

## Slide 4
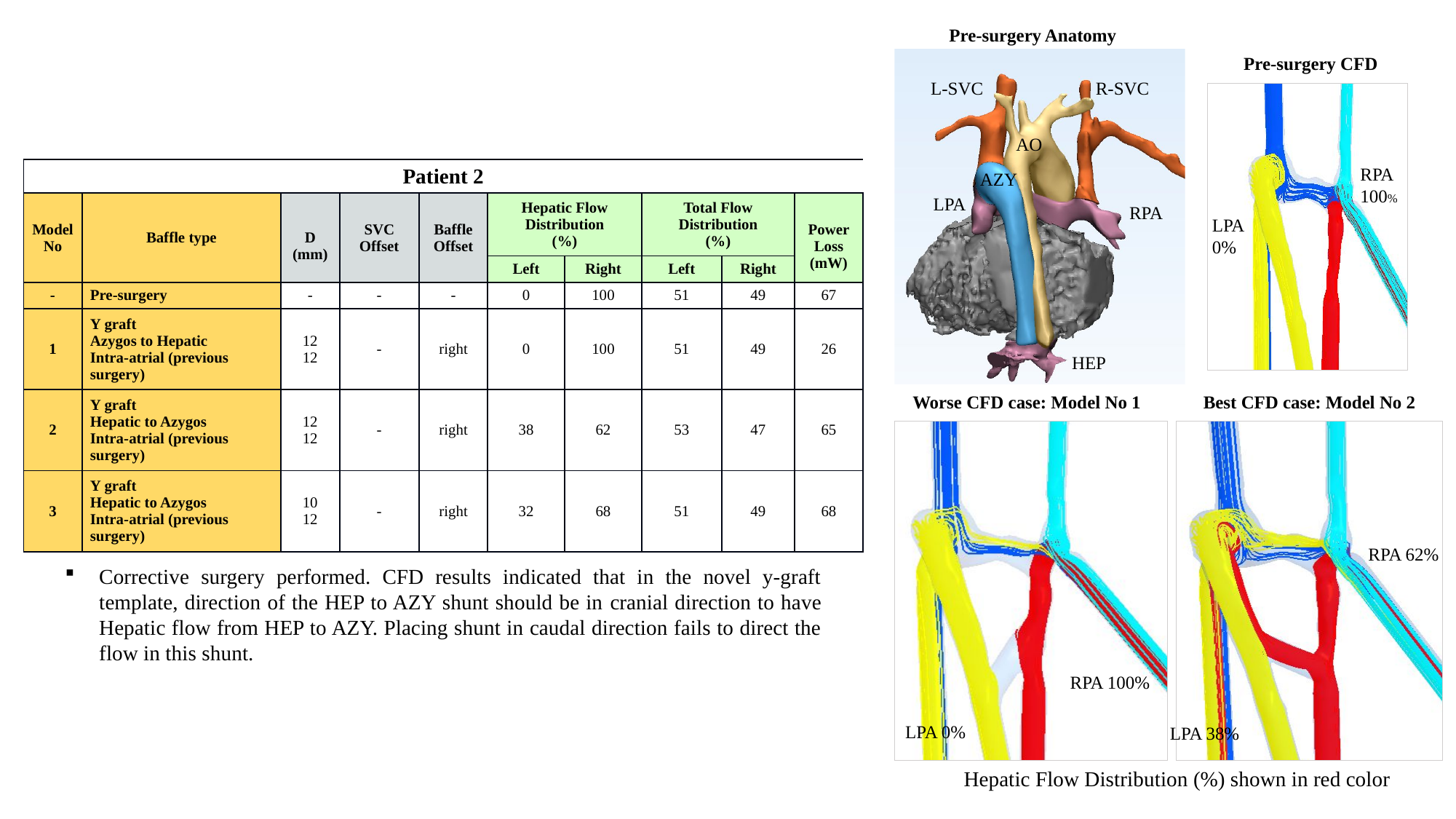

Pre-surgery Anatomy
R-SVC
L-SVC
AO
AZY
LPA
RPA
HEP
Pre-surgery CFD
RPA
100%
LPA
0%
| Patient 2 | Patient 3 | | | | | | | | |
| --- | --- | --- | --- | --- | --- | --- | --- | --- | --- |
| Model No | Baffle type | D (mm) | SVC Offset | Baffle Offset | Hepatic Flow Distribution (%) | | Total Flow Distribution (%) | | Power Loss (mW) |
| | | | | | Left | Right | Left | Right | |
| - | Pre-surgery | - | - | - | 0 | 100 | 51 | 49 | 67 |
| 1 | Y graft Azygos to Hepatic Intra-atrial (previous surgery) | 12 12 | - | right | 0 | 100 | 51 | 49 | 26 |
| 2 | Y graft Hepatic to Azygos Intra-atrial (previous surgery) | 12 12 | - | right | 38 | 62 | 53 | 47 | 65 |
| 3 | Y graft Hepatic to Azygos Intra-atrial (previous surgery) | 10 12 | - | right | 32 | 68 | 51 | 49 | 68 |
Best CFD case: Model No 2
RPA 62%
LPA 38%
Worse CFD case: Model No 1
RPA 100%
LPA 0%
Corrective surgery performed. CFD results indicated that in the novel y-graft template, direction of the HEP to AZY shunt should be in cranial direction to have Hepatic flow from HEP to AZY. Placing shunt in caudal direction fails to direct the flow in this shunt.
Hepatic Flow Distribution (%) shown in red color

## Slide 5
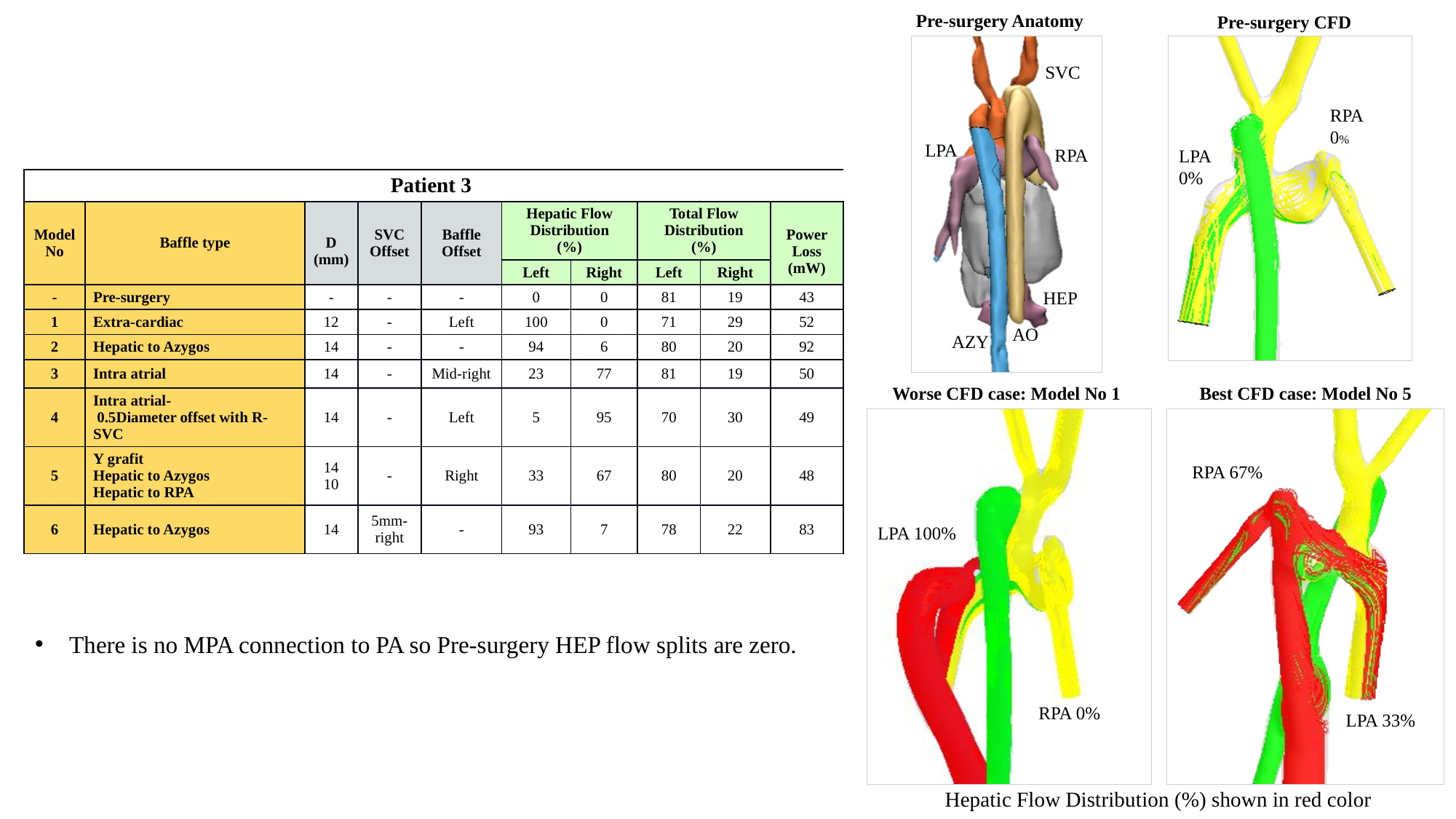

Pre-surgery Anatomy
SVC
LPA
RPA
AO
AZY
HEP
Pre-surgery CFD
RPA
0%
LPA
0%
| Patient 3 | Patient 3 | | | | | | | | |
| --- | --- | --- | --- | --- | --- | --- | --- | --- | --- |
| Model No | Baffle type | D (mm) | SVC Offset | Baffle Offset | Hepatic Flow Distribution (%) | | Total Flow Distribution (%) | | Power Loss (mW) |
| | | | | | Left | Right | Left | Right | |
| - | Pre-surgery | - | - | - | 0 | 0 | 81 | 19 | 43 |
| 1 | Extra-cardiac | 12 | - | Left | 100 | 0 | 71 | 29 | 52 |
| 2 | Hepatic to Azygos | 14 | - | - | 94 | 6 | 80 | 20 | 92 |
| 3 | Intra atrial | 14 | - | Mid-right | 23 | 77 | 81 | 19 | 50 |
| 4 | Intra atrial- 0.5Diameter offset with R-SVC | 14 | - | Left | 5 | 95 | 70 | 30 | 49 |
| 5 | Y grafit Hepatic to Azygos Hepatic to RPA | 14 10 | - | Right | 33 | 67 | 80 | 20 | 48 |
| 6 | Hepatic to Azygos | 14 | 5mm-right | - | 93 | 7 | 78 | 22 | 83 |
Worse CFD case: Model No 1
LPA 100%
RPA 0%
Best CFD case: Model No 5
RPA 67%
LPA 33%
There is no MPA connection to PA so Pre-surgery HEP flow splits are zero.
Hepatic Flow Distribution (%) shown in red color

## Slide 6
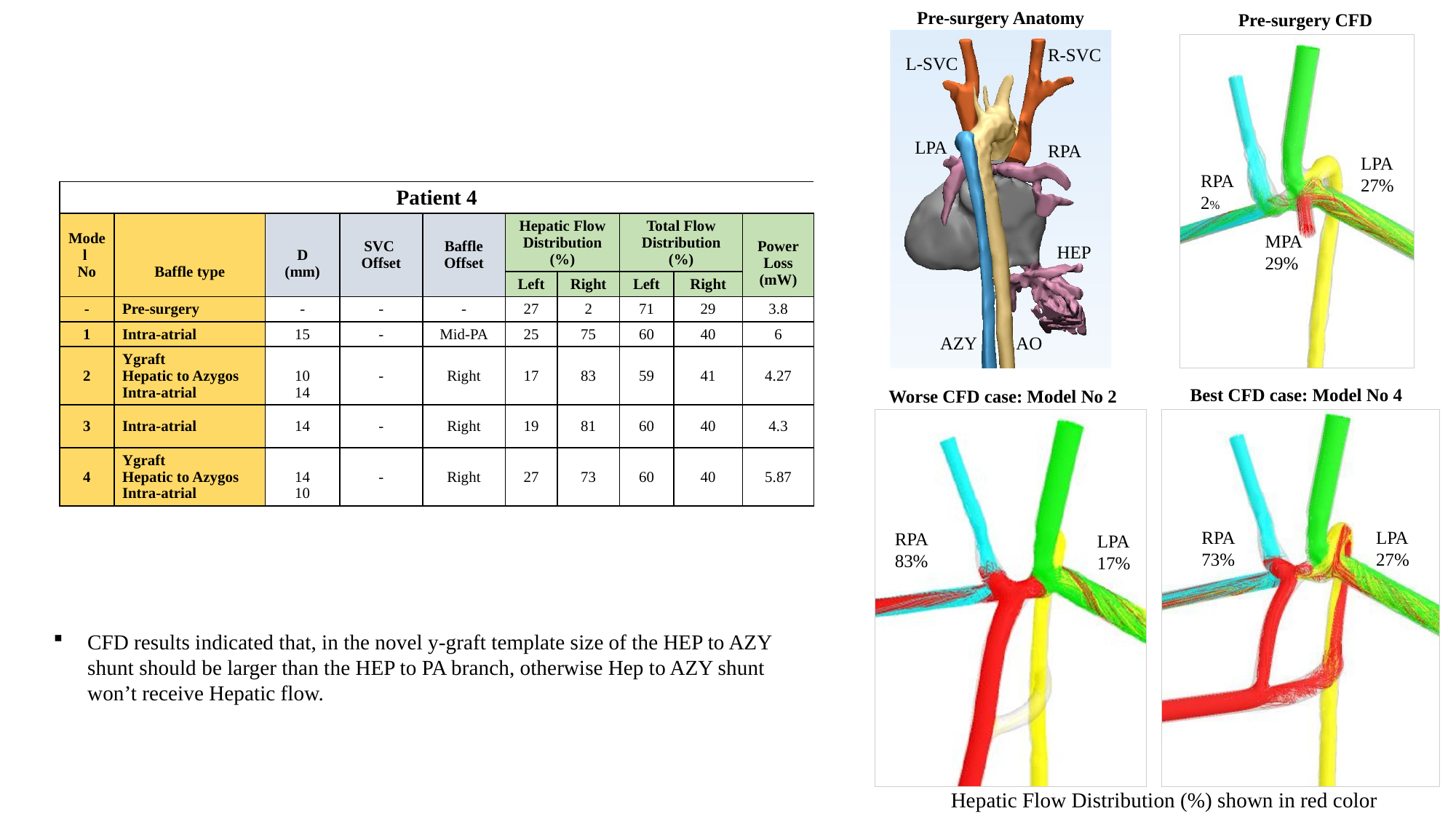

Pre-surgery Anatomy
R-SVC
L-SVC
LPA
RPA
AO
AZY
HEP
Pre-surgery CFD
LPA
27%
RPA
2%
MPA
29%
| Patient 4 | Patient 4 | | | | | | | | |
| --- | --- | --- | --- | --- | --- | --- | --- | --- | --- |
| Model No | Baffle type | D (mm) | SVC Offset | Baffle Offset | Hepatic Flow Distribution (%) | | Total Flow Distribution (%) | | Power Loss (mW) |
| | | | | | Left | Right | Left | Right | |
| - | Pre-surgery | - | - | - | 27 | 2 | 71 | 29 | 3.8 |
| 1 | Intra-atrial | 15 | - | Mid-PA | 25 | 75 | 60 | 40 | 6 |
| 2 | Ygraft Hepatic to Azygos Intra-atrial | 10 14 | - | Right | 17 | 83 | 59 | 41 | 4.27 |
| 3 | Intra-atrial | 14 | - | Right | 19 | 81 | 60 | 40 | 4.3 |
| 4 | Ygraft Hepatic to Azygos Intra-atrial | 14 10 | - | Right | 27 | 73 | 60 | 40 | 5.87 |
Best CFD case: Model No 4
RPA
73%
LPA
27%
Worse CFD case: Model No 2
RPA
83%
LPA
17%
CFD results indicated that, in the novel y-graft template size of the HEP to AZY shunt should be larger than the HEP to PA branch, otherwise Hep to AZY shunt won’t receive Hepatic flow.
Hepatic Flow Distribution (%) shown in red color

## Slide 7
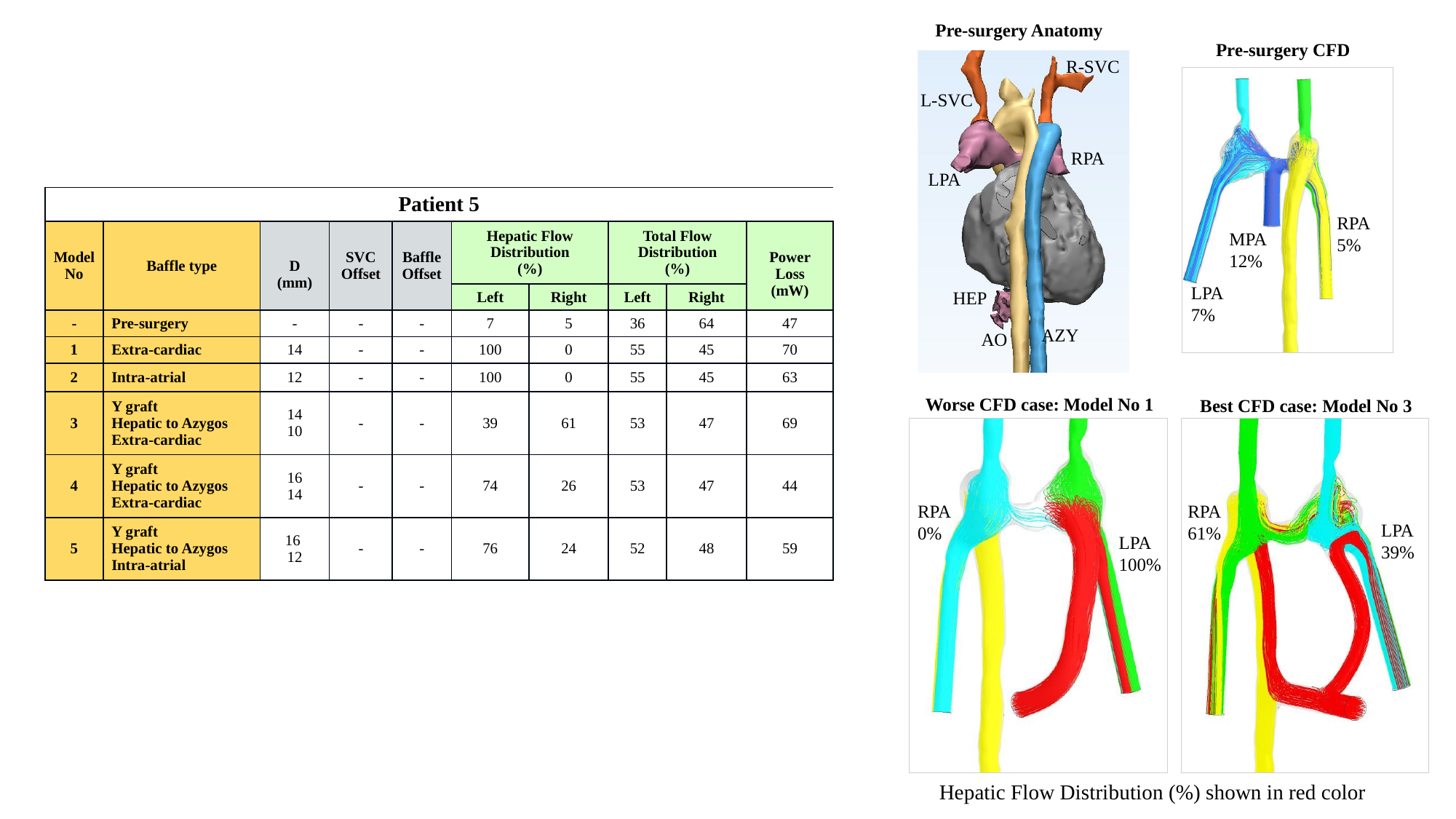

Pre-surgery Anatomy
R-SVC
L-SVC
RPA
LPA
AZY
AO
HEP
Pre-surgery CFD
RPA 5%
MPA 12%
LPA 7%
| Patient 5 | Patient 3 | | | | | | | | |
| --- | --- | --- | --- | --- | --- | --- | --- | --- | --- |
| Model No | Baffle type | D (mm) | SVC Offset | Baffle Offset | Hepatic Flow Distribution (%) | | Total Flow Distribution (%) | | Power Loss (mW) |
| | | | | | Left | Right | Left | Right | |
| - | Pre-surgery | - | - | - | 7 | 5 | 36 | 64 | 47 |
| 1 | Extra-cardiac | 14 | - | - | 100 | 0 | 55 | 45 | 70 |
| 2 | Intra-atrial | 12 | - | - | 100 | 0 | 55 | 45 | 63 |
| 3 | Y graft Hepatic to Azygos Extra-cardiac | 14 10 | - | - | 39 | 61 | 53 | 47 | 69 |
| 4 | Y graft Hepatic to Azygos Extra-cardiac | 16 14 | - | - | 74 | 26 | 53 | 47 | 44 |
| 5 | Y graft Hepatic to Azygos Intra-atrial | 16 12 | - | - | 76 | 24 | 52 | 48 | 59 |
Worse CFD case: Model No 1
RPA
0%
LPA
100%
Best CFD case: Model No 3
RPA
61%
LPA
39%
Hepatic Flow Distribution (%) shown in red color

## Slide 8
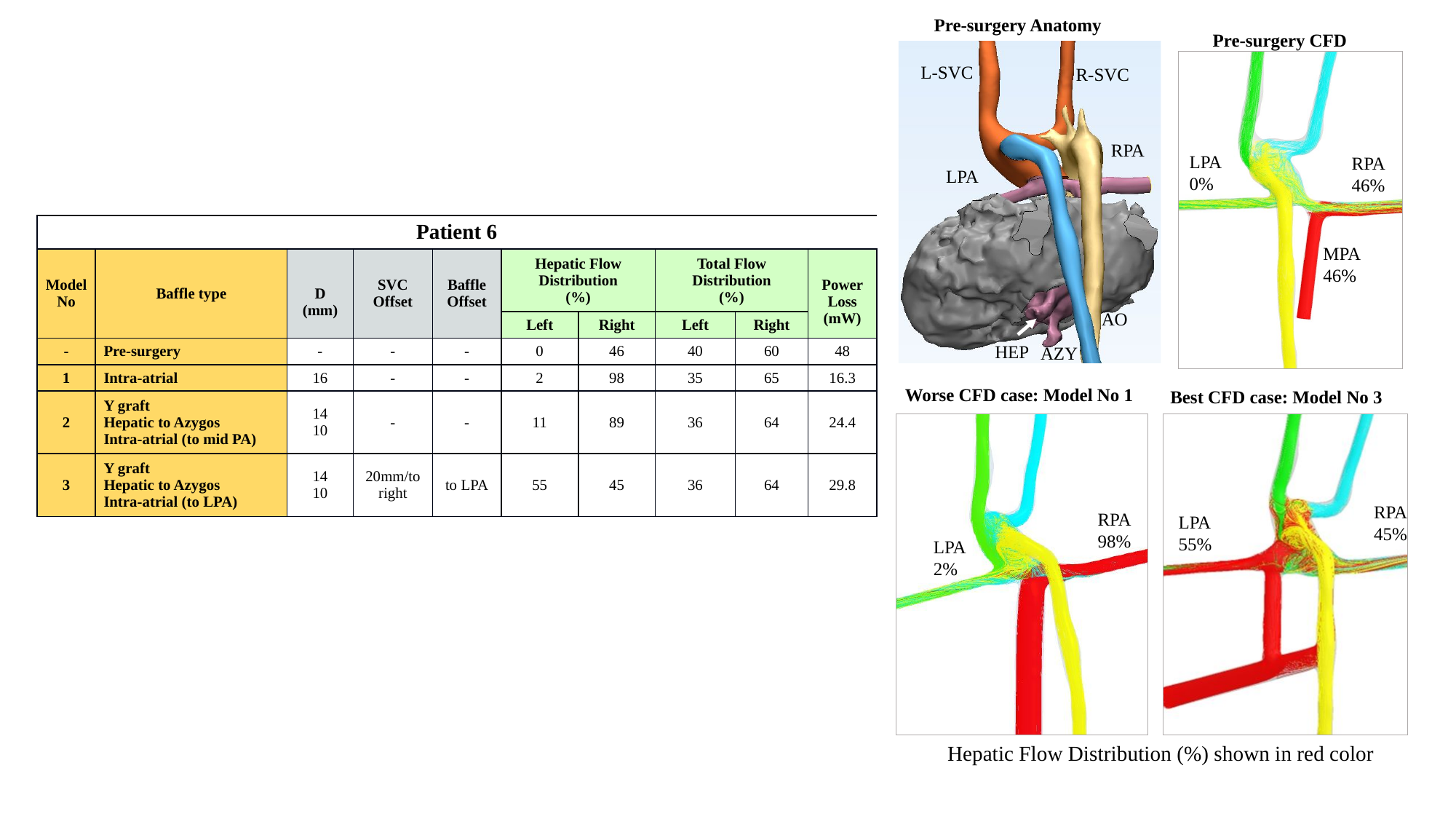

Pre-surgery Anatomy
L-SVC
R-SVC
RPA
LPA
AO
AZY
HEP
Pre-surgery CFD
LPA 0%
RPA 46%
MPA 46%
| Patient 6 | Patient 3 | | | | | | | | |
| --- | --- | --- | --- | --- | --- | --- | --- | --- | --- |
| Model No | Baffle type | D (mm) | SVC Offset | Baffle Offset | Hepatic Flow Distribution (%) | | Total Flow Distribution (%) | | Power Loss (mW) |
| | | | | | Left | Right | Left | Right | |
| - | Pre-surgery | - | - | - | 0 | 46 | 40 | 60 | 48 |
| 1 | Intra-atrial | 16 | - | - | 2 | 98 | 35 | 65 | 16.3 |
| 2 | Y graft Hepatic to Azygos Intra-atrial (to mid PA) | 14 10 | - | - | 11 | 89 | 36 | 64 | 24.4 |
| 3 | Y graft Hepatic to Azygos Intra-atrial (to LPA) | 14 10 | 20mm/to right | to LPA | 55 | 45 | 36 | 64 | 29.8 |
Worse CFD case: Model No 1
RPA
98%
LPA
2%
Best CFD case: Model No 3
RPA
45%
LPA
55%
Hepatic Flow Distribution (%) shown in red color

## Slide 9
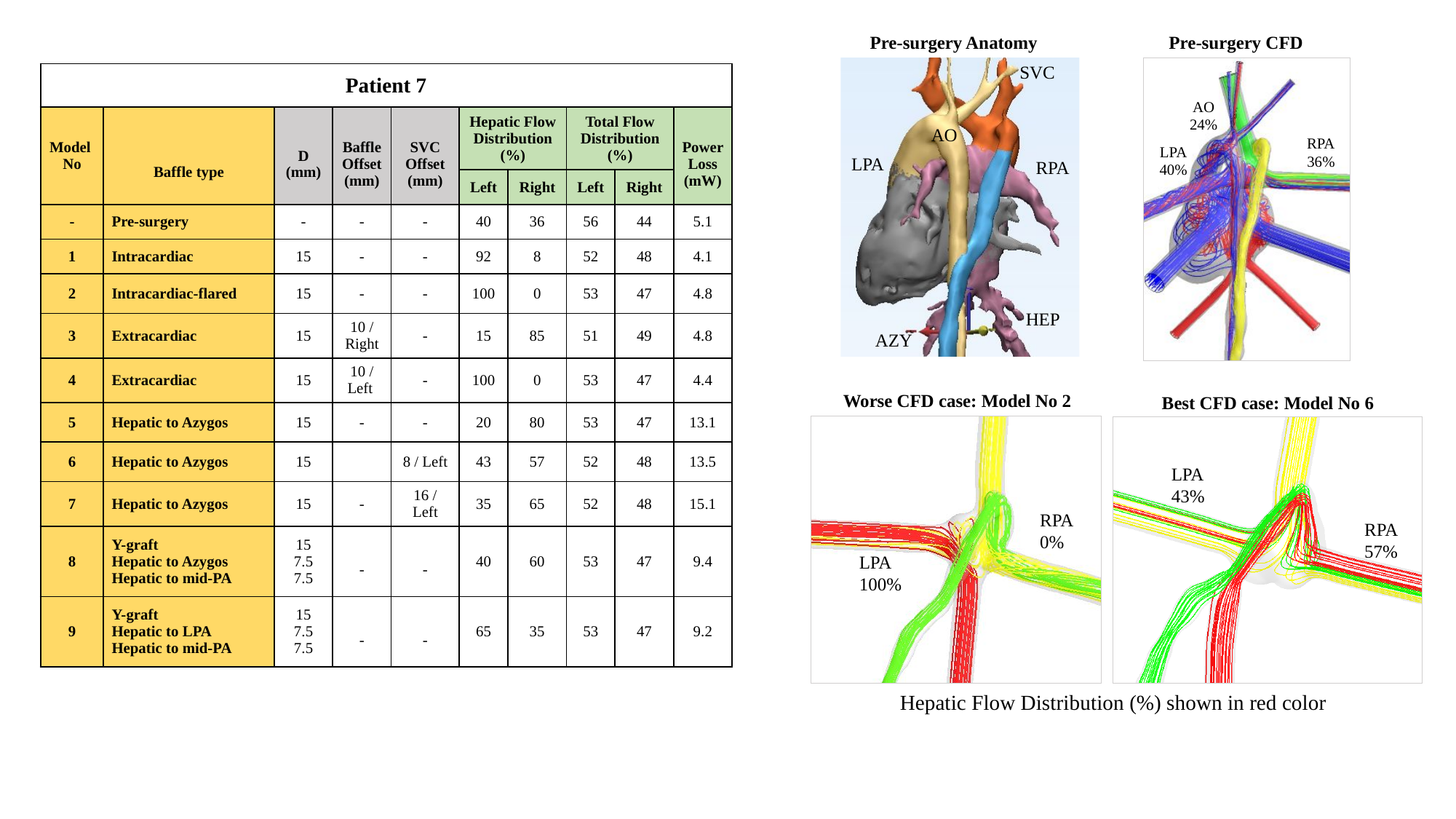

Pre-surgery Anatomy
SVC
AO
LPA
RPA
AZY
HEP
Pre-surgery CFD
AO
24%
RPA
36%
LPA
40%
| Patient 7 | Patient 2 | | | | | | | | |
| --- | --- | --- | --- | --- | --- | --- | --- | --- | --- |
| Model No | Baffle type | D (mm) | Baffle Offset (mm) | SVC Offset (mm) | Hepatic Flow Distribution (%) | | Total Flow Distribution (%) | | Power Loss (mW) |
| | | | | | Left | Right | Left | Right | |
| - | Pre-surgery | - | - | - | 40 | 36 | 56 | 44 | 5.1 |
| 1 | Intracardiac | 15 | - | - | 92 | 8 | 52 | 48 | 4.1 |
| 2 | Intracardiac-flared | 15 | - | - | 100 | 0 | 53 | 47 | 4.8 |
| 3 | Extracardiac | 15 | 10 / Right | - | 15 | 85 | 51 | 49 | 4.8 |
| 4 | Extracardiac | 15 | 10 / Left | - | 100 | 0 | 53 | 47 | 4.4 |
| 5 | Hepatic to Azygos | 15 | - | - | 20 | 80 | 53 | 47 | 13.1 |
| 6 | Hepatic to Azygos | 15 | | 8 / Left | 43 | 57 | 52 | 48 | 13.5 |
| 7 | Hepatic to Azygos | 15 | - | 16 / Left | 35 | 65 | 52 | 48 | 15.1 |
| 8 | Y-graft Hepatic to Azygos Hepatic to mid-PA | 15 7.5 7.5 | - | - | 40 | 60 | 53 | 47 | 9.4 |
| 9 | Y-graft Hepatic to LPA Hepatic to mid-PA | 15 7.5 7.5 | - | - | 65 | 35 | 53 | 47 | 9.2 |
Worse CFD case: Model No 2
RPA
0%
LPA
100%
Best CFD case: Model No 6
LPA
43%
RPA
57%
Hepatic Flow Distribution (%) shown in red color

## Slide 10
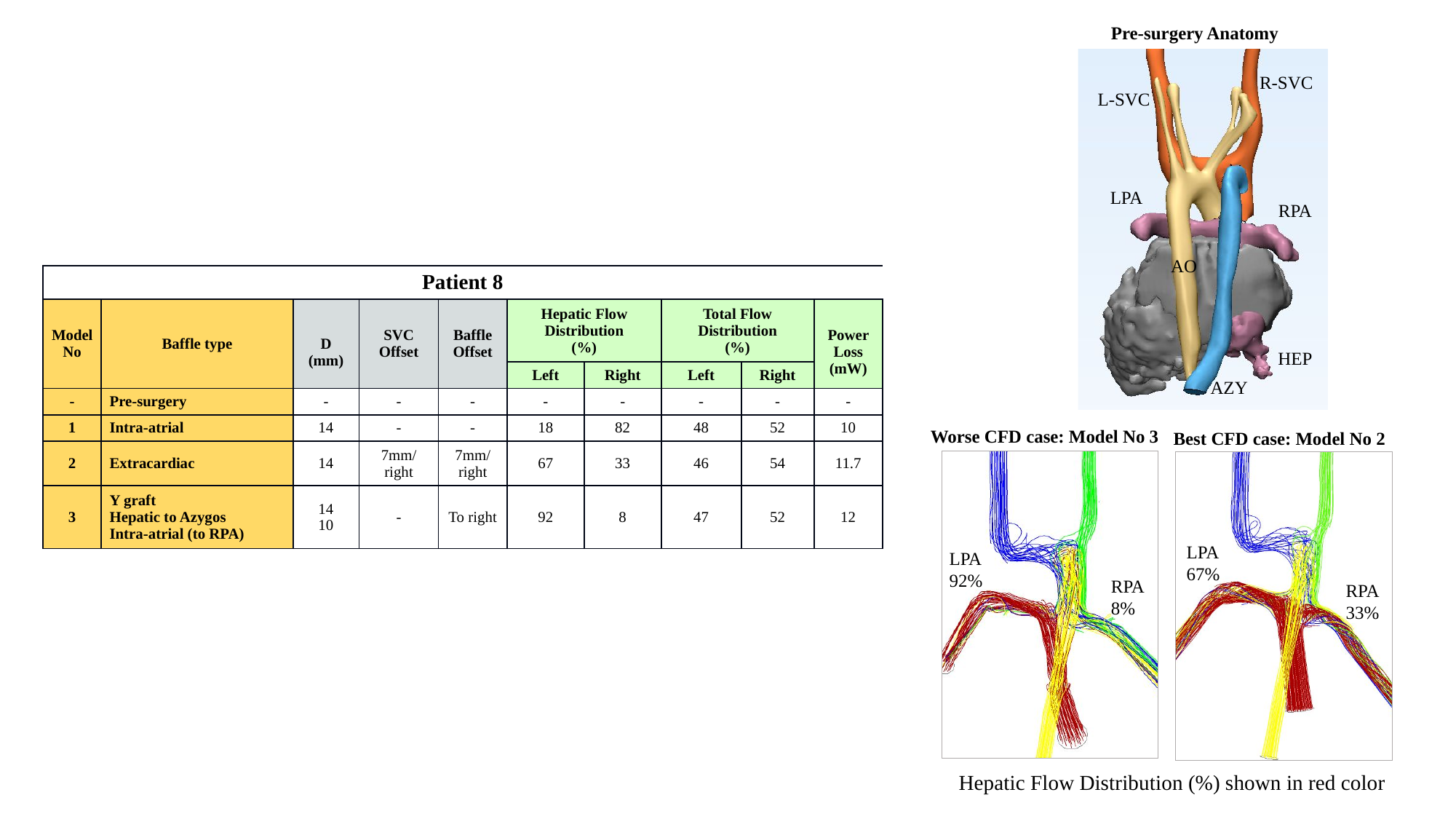

Pre-surgery Anatomy
R-SVC
L-SVC
LPA
RPA
AO
AZY
HEP
| Patient 8 | Patient 3 | | | | | | | | |
| --- | --- | --- | --- | --- | --- | --- | --- | --- | --- |
| Model No | Baffle type | D (mm) | SVC Offset | Baffle Offset | Hepatic Flow Distribution (%) | | Total Flow Distribution (%) | | Power Loss (mW) |
| | | | | | Left | Right | Left | Right | |
| - | Pre-surgery | - | - | - | - | - | - | - | - |
| 1 | Intra-atrial | 14 | - | - | 18 | 82 | 48 | 52 | 10 |
| 2 | Extracardiac | 14 | 7mm/ right | 7mm/ right | 67 | 33 | 46 | 54 | 11.7 |
| 3 | Y graft Hepatic to Azygos Intra-atrial (to RPA) | 14 10 | - | To right | 92 | 8 | 47 | 52 | 12 |
Worse CFD case: Model No 3
LPA
92%
RPA
8%
Best CFD case: Model No 2
LPA
67%
RPA
33%
Hepatic Flow Distribution (%) shown in red color

## Slide 11
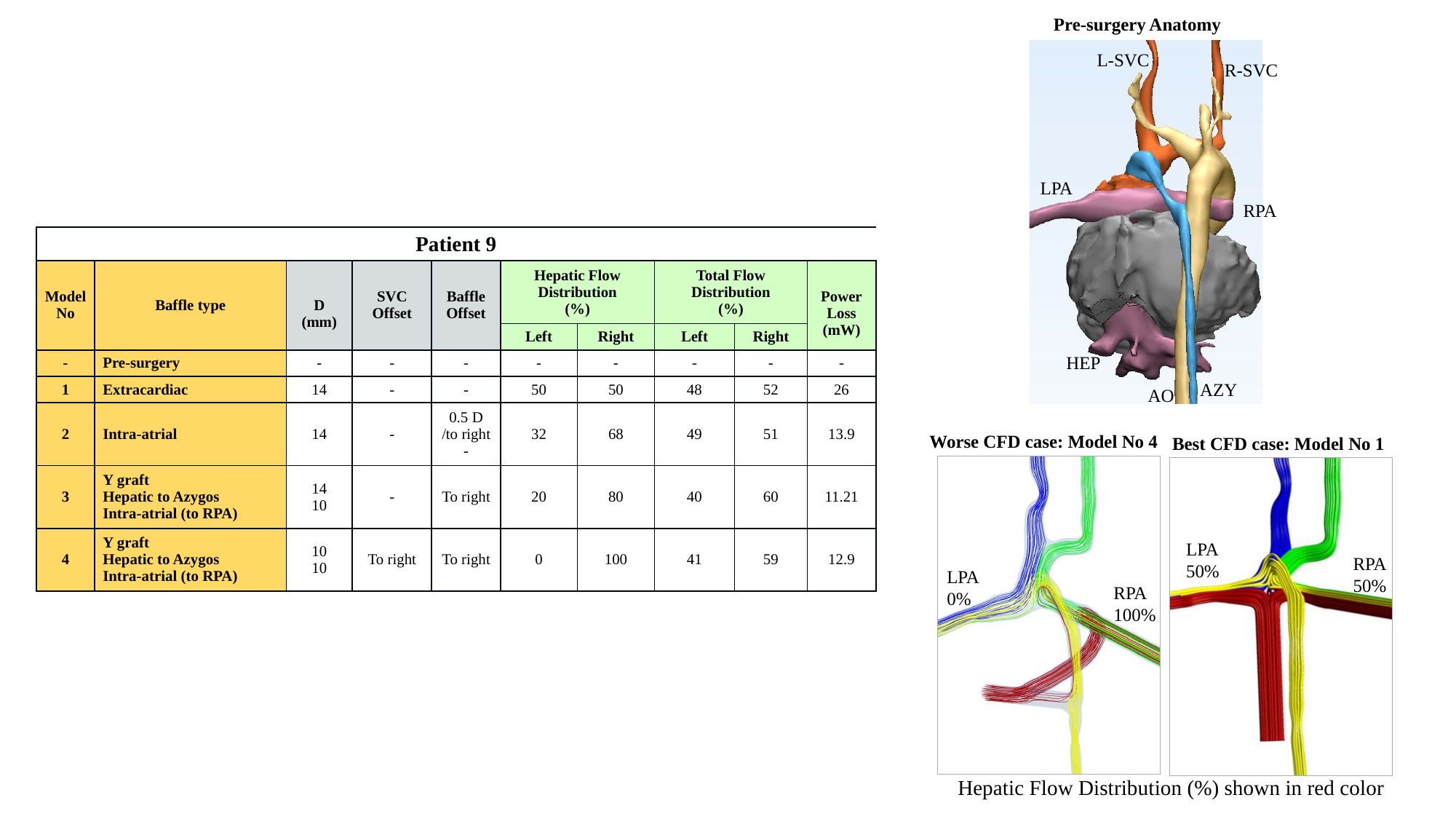

Pre-surgery Anatomy
L-SVC
R-SVC
LPA
RPA
AZY
AO
HEP
| Patient 9 | Patient 3 | | | | | | | | |
| --- | --- | --- | --- | --- | --- | --- | --- | --- | --- |
| Model No | Baffle type | D (mm) | SVC Offset | Baffle Offset | Hepatic Flow Distribution (%) | | Total Flow Distribution (%) | | Power Loss (mW) |
| | | | | | Left | Right | Left | Right | |
| - | Pre-surgery | - | - | - | - | - | - | - | - |
| 1 | Extracardiac | 14 | - | - | 50 | 50 | 48 | 52 | 26 |
| 2 | Intra-atrial | 14 | - | 0.5 D /to right - | 32 | 68 | 49 | 51 | 13.9 |
| 3 | Y graft Hepatic to Azygos Intra-atrial (to RPA) | 14 10 | - | To right | 20 | 80 | 40 | 60 | 11.21 |
| 4 | Y graft Hepatic to Azygos Intra-atrial (to RPA) | 10 10 | To right | To right | 0 | 100 | 41 | 59 | 12.9 |
Worse CFD case: Model No 4
LPA
0%
RPA
100%
Best CFD case: Model No 1
LPA
50%
RPA
50%
Hepatic Flow Distribution (%) shown in red color
