# Supplementary material for: A novel Fontan Y-graft for interrupted inferior vena cava and azygos continuation
Source: Interact Cardiovasc Thorac Surg. 2022 Feb 3;34(6):1095–105. doi: 10.1093/icvts/ivac001 (PMC9159461; doi:10.1093/icvts/ivac001)
Supplement: ivac001_Supplementary_Data [file ivac001_supplementary_data.zip › ivac001-suppl_data/Supplementary Material 2revised.docx]

**­­A novel** **Fontan Y-graft for the interrupted inferior**

**vena cava and azygous continuation**

**Supplementary Material 2:** MRI and PC-MRI protocols are provided. In addition, details of in vitro experiments used for model validation is included.

**MRI Imaging:** The first case (Patient 1) was a 6-year-old male patient who had left atrial isomerism, double outlet right ventricle, double SVC, moderate pulmonic stenosis, interrupted IVC with AZY continuation to the left SVC branch (L-SVC). Furthermore, the presence of R-SVC created significant challenges to surgically distribute equal hepatic flow to both lungs. The second case (Patient 2) was a 14-year-old female single ventricle patient, who had a cavopulmonary connection on the right pulmonary artery (RPA) and then a Kawashima procedure consisting of a superior cavopulmonary connection on the left pulmonary artery (LPA) performed six years earlier at the age of 8 years. In this patient, antegrade pulmonary flow was preserved to prevent PAVF formation by distributing hepatic venous blood to both lungs. Preserving the antegrade flow may have been considered as a safe surgical approach, but oxygen saturation decreased to 69% and left PAVFs developed (as shown in Figure 1A) six years after the Kawashima procedure was performed. The low oxygen saturation was due to a failed single extracardiac shunt requiring corrective re-do surgery to change the graft configuration. These clinical characteristics made Patient 2 a good candidate to study the key geometric features of the new Y-graft surgical template.

All nine patients underwent a thoracal magnetic resonance angiography (MRA) with cardiac perfusion by 3.0 Tesla scanner (Philips Ingenia; Philips Medical Systems, Best, NL) with 0.2 mmol/kg of gadolinium-based contrast media. The images were acquired during one breath-hold. The raw image data was processed by medical image processing software Mimics Innovation Suite 17 (Materialise, Leuven, Belgium) and 3D Slicer open-source application for segmentation of the region of interest. The heart cavity and aorta were also included in the pre-surgery models to provide landmarks for surgical planning.

For post-operative verification of the intended surgical template, patients underwent MRA scanning within one year of surgery, without contrast agent administration. PA flow assessment and 3D reconstructions were performed. Image slices were acquired during one breath-hold of 12 seconds with retrospective gating and 20 cardiac phases. Flow conditions were measured using the diagnostic catheter angiography gradients of the patient. In post-operative models, PA, AZY, HEP and SVC reconstructions were included in the geometry.

***In Vitro* validation test, Hepatic flow measurement through dye concentrations**

In order to compare the hemodynamic performance of surgical alternatives, computational fluid dynamics (CFD) with lumped peripheral vascular resistance boundary conditions were used.

The three‐dimensional Navier‐Stokes equations were solved in Fluent (Ansys Inc., Canonsburg, PA) with a transient pressure‐based solver. The pressure‐velocity coupling algorithm was set as Pressure-Implicit with Splitting of Operators (PISO) algorithm. Second-order accuracy in both pressure and velocity was used in the discretization of the Navier-Stokes equations. Blood is considered as Newtonian fluid. Resistance boundary conditions were used at all outlets. The resistance values were obtained from pre-surgery MRA data and applied on all virtual surgery models. The post-surgery model inlet velocities and resistance values were obtained from post-operative data.

To validate the computational upshots, a bench-top experiment was performed for the Patient 7 (refer to Table 1 in the main manuscript). The patient was a nine-years-old female with single ventricle, interrupted IVC and Azygos continuation. She had undergone Glenn already and Fontan procedure is performed at the current stage. MRI scan that was used for the anatomical reconstruction was performed at the Siyami Ersek Hospital Istanbul, Turkey in 2017. A virtual surgery was performed on the reconstructed presurgical model. A 15mm diameter graft was connected to the middle of the Pulmonary artery in virtual reconstruction to represent the Fontan graft. The virtually repaired anatomy was used in the in vitro test.

The experimental validation was carried out for the scaled anatomical geometry (scale factor = 0.4) while keeping the clinical flow conditions identical through non-dimensional analysis and similitude. The scaled model was realized using a stereolithography 3D printer (Formlabs, Somerville, Massachusetts, US) following a post-curing process to enhance the model rigidity and reduce the water absorption (Supplementary Figure 1). The orientation of the prototype model in the experimental setup was carefully adjusted to keep the gravity acceleration normal to the IVC inlet. Supplementary Table 1 summarizes the boundary condition types and values used in the experimental validation.

Distilled water was used as the working fluid during the experiments. Quantification of the hepatic flow distribution was achieved by introducing a tracer fluid (Pelikan Royal blue ink) into the IVC inlet with known concentration. Subsequently, two volume samples were collected from the outlets (RPA and LPA) for a control time of 180 s and the concentration was measured using Multiscan FC microplate reader (ThermoFisher, Waltham, Massachusetts, US). Finally, the hepatic flow distribution was quantified by satisfying the mass conservation equation for the tracer fluid. All measurement values from two experiments are provided in Supplementary Table 2. The averaged values of the experiments together with the hepatic flow distribution and pulmonary flow split are presented in Supplementary Table 3.

Computational model of the experimental case was built and solved using the flow solver explained in Section 2.3 of the main text. Same working fluid specifications and boundary conditions as in vitro test were assigned in the computational simulation. Hepatic and total pulmonary flow splits computed from the CFD model are compared to the experimental results and found to be within reasonable agreement having a maximum of ~10% error (Supplementary Table 4).


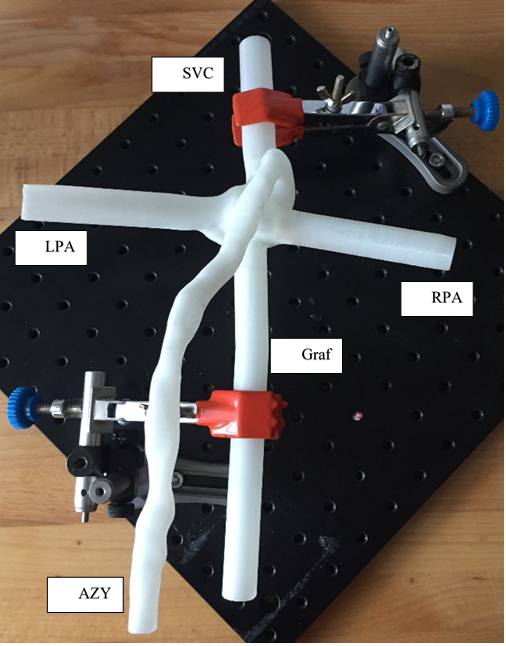


**Supplementary Figure 1:** 3D printed anatomical model of Patient 7 (Table 1) after virtual surgery using 15mm diameter graft. This case is one of the trial surgical configurations for virtual surgery and is not used in the real surgery of Patient 7.

**Supplementary Table 1**: Boundary condition types and values used during the experimental validation. IVC: Inferior vena cava, SVC: Superior vena cava, AZY: Azygous continuation, RPA: Right pulmonary artery, LPA: Left pulmonary artery, LPH: Liter per hour.

|  | **BC type** | **BC value** |
| --- | --- | --- |
| **IVC** | Inlet flowrate | 36 LPH |
| **SVC** | Inlet flowrate | 45 LPH |
| **AZY** | Inlet flowrate | 15 LPH |
| **RPA** | Pressure outlet | 0 Gauge |
| **LPA** | Pressure outlet | 0 Gauge |

**Supplementary Table 2**: Measured concentration and flowrates for two validation experiments. N.Concentration represents the concentration normalized by the IVC concentration. v: Tracer fluid volume, V: total solution volume. Here in the concentration formula the numerator is the tracer fluid volume and when there is no tracer fluid in the solution the numerator is zero which results in the division to be zero.

|  | **First Experiment** | | | **Second Experiment** | | |
| --- | --- | --- | --- | --- | --- | --- |
|  | **Flowrate (LPH)** | **Concentration (‰ v/V)** | **N.Concentration** | **Flowrate (LPH)** | **Concentration (‰ v/V)** | **N.Concentration** |
| **IVC** | 36 | 5.5 | 1 | 36 | 5.5 | 1 |
| **SVC** | 45 | 0 | 0 | 45 | 0 | 0 |
| **AZY** | 15 | 0 | 0 | 15 | 0 | 0 |
| **LPA** | 51.3 | 3.17 | 0.5764 | 49.2 | 3.18 | 0.5787 |
| **RPA** | 46.2 | 0.6385 | 0.116 | 47.2 | 0.7472 | 0.1359 |

**Supplementary Table 3**: Averaged measurement data from two validation experiments. N.Concentration: concentration normalized by the IVC concentration, HFD: hepatic flow distribution, PFS: pulmonary flow split.

|  | **Flowrate (LPH)** | **N.Concentration** | **PFS (%)** | **HFD (%)** |
| --- | --- | --- | --- | --- |
| **IVC** | 36 | 1 | ---------- | ---------- |
| **SVC** | 45 | 0 | ---------- | ---------- |
| **AZY** | 15 | 0 | ---------- | ---------- |
| **LPA** | 50.25 | 0.58 | 52 | 83 |
| **RPA** | 46.7 | 0.13 | 48 | 17 |

**Supplementary Table 4**: Comparison of *in vitro* test measurements with the computed flow splits in CFD model. RPA/LPA flow ratios correspond to their % shares of total pulmonary flow.

|  | **Hepatic Flow Distribution (HFD)** | | | **Pulmonary Flow Split (PFS)** | | |
| --- | --- | --- | --- | --- | --- | --- |
|  | **LPA (%)** | **RPA (%)** | **RPA/LPA** | **LPA (%)** | **RPA (%)** | **RPA/LPA** |
| **CFD** | 85 | 15 | 18 | 54 | 46 | 85 |
| ***In-Vitro* test** | 83 | 17 | 20 | 52 | 48 | 92 |
| **Error (%)** | - | - | 10 | - | - | 8 |
