# Supplementary material for: A novel Fontan Y-graft for interrupted inferior vena cava and azygos continuation
Source: Interact Cardiovasc Thorac Surg. 2022 Feb 3;34(6):1095–105. doi: 10.1093/icvts/ivac001 (PMC9159461; doi:10.1093/icvts/ivac001)
Supplement: ivac001_Supplementary_Data [file ivac001_supplementary_data.zip › ivac001-suppl_data/Supplementary Material 3.docx]

**­­A novel** **Fontan Y-graft for the interrupted inferior**

**vena cava and azygous continuation**

**Supplementary Material** 3: Discussion on the *potential* long-term hemodynamic function of the vascular resistance network of the proposed surgical template

Hemodynamic design of the TCPC site is discussed in this section. In Figure 1 the circuit diagram of the proposed surgical configuration is illustrated demonstrating the potential for further conduit resistance drop as vessels grow. This figure shows the fixed (T_tcpc_ and R_graft_) and variable flow resistance (R_AZY_). Variable resistance, R_AZY_ will decrease due to potential vessel growth post-operatively either due to somatic or flow-induced growth. This diagram shows that in our design, at early post-op, the resistance bottleneck is not the fixed shunt, i.e., R_graft_ as we specifically make its diameter as large as possible (initially, early post-op, R_AZY_>R_graft_). The bottleneck is due to the azygous vessel resistance, but it can grow and has the potential to decrease the power loss of the system significantly. It is intended that R_AZY_ will almost become equal to R_tcpc_ and R_graft_. Presumed *growth simulations showed that if this condition is reached the hepatic flow distribution has potential to be more balanced than the early post-op.*

**

**Figure 1:** A sketch of the lumped primary resistances of the Fontan circuit is shown. R_AZY_ is the flow resistance of the native azygous branch which will decrease due to *possible* somatic, and *possible* flow induced lumen growth during the post-operative course. The other resistances are artificial “fixed” resistances. To benefit the *potential* growth capability of the AZY branch the diameter associated with R_graft_ is kept as large as possible. This condition is simulated in our patient specific analysis as long-term post-operative state (Table 2). Resistances of the junctions, though important, are ignored for clarity.

While this feature may appear to be an important feature of the proposed surgical design, it requires that the presumed growth potential actually occurs for the individual patient. This is not yet fully proven in this manuscript due to the short post-op follow-up, even though the native vessel growth has been demonstrated in other studies. This desired effect may well emerge after several years. During that time the balanced hepatic flow to both lungs may be reduced or altered due to other influencing factors, not accounted in this idealized hemodynamic scenario.
