# Supplementary material for: A novel Fontan Y-graft for interrupted inferior vena cava and azygos continuation
Source: Interact Cardiovasc Thorac Surg. 2022 Feb 3;34(6):1095–105. doi: 10.1093/icvts/ivac001 (PMC9159461; doi:10.1093/icvts/ivac001)
Supplement: ivac001_Supplementary_Data [file ivac001_supplementary_data.zip › ivac001-suppl_data/Supplementary Material 4_video_description.docx]

**Supplementary Material 4 - Video legend**

Video illustrates the pre-operative Angiography recordings for Patient 6 (Table 1). PAVMs were observed in the left lung before surgery. In surgical planning stage, virtual surgery and CFD analysis were performed, and the novel Y-graft template was selected for the real surgery, securing balanced Hepatic and pulmonary venous flow to both lungs. Post-operative Angiography recordings verified desired hemodynamic performance of the novel Y-graft. PAVMs had disappeared within one-year after surgery.
